# Optimal bioenergy power generation for climate change mitigation with or without carbon sequestration

**DOI:** 10.1038/ncomms13160

**Published:** 2016-10-21

**Authors:** Dominic Woolf, Johannes Lehmann, David R. Lee

**Affiliations:** 1 Soil and Crop Sciences, School of Integrative Plant Science, Cornell University, Ithaca, New York 14853, USA; 2 Atkinson Center for a Sustainable Future, Cornell University, Ithaca, New York 14853, USA; 3 Charles H. Dyson School of Applied Economics and Management, Cornell University, Ithaca, New York 14853, USA

## Abstract

Restricting global warming below 2 °C to avoid catastrophic climate change will require atmospheric carbon dioxide removal (CDR). Current integrated assessment models (IAMs) and Intergovernmental Panel on Climate Change scenarios assume that CDR within the energy sector would be delivered using bioenergy with carbon capture and storage (BECCS). Although bioenergy-biochar systems (BEBCS) can also deliver CDR, they are not included in any IPCC scenario. Here we show that despite BECCS offering twice the carbon sequestration and bioenergy per unit biomass, BEBCS may allow earlier deployment of CDR at lower carbon prices when long-term improvements in soil fertility offset biochar production costs. At carbon prices above $1,000 Mg^−1^ C, BECCS is most frequently (*P*>0.45, calculated as the fraction of Monte Carlo simulations in which BECCS is the most cost effective) the most economic biomass technology for climate-change mitigation. At carbon prices below $1,000 Mg^−1^ C, BEBCS is the most cost-effective technology only where biochar significantly improves agricultural yields, with pure bioenergy systems being otherwise preferred.

## Abstract (web summary)

Prior mitigation assessments of atmospheric CO_2_ removal rely on bioenergy carbon capture and storage (BECCS), excluding bioenergy-biochar systems (BEBCS). Here, Woolf *et al*. find that BEBCS offers an alternative cost-effective solution, and may allow earlier CO_2_ removal at a lower carbon price.

A target rise in global mean temperature of less than 2 °C has been widely advocated as necessary to prevent dangerous anthropogenic interference with the climate system^1^,^2^. Most mitigation scenarios that achieve this include widespread carbon dioxide removal (CDR) in the second half of the century to compensate for residual emissions from sectors where mitigation is costly and to recover from an overshoot of CO_2_ emissions^3^. Therefore, CDR is critical to achieving climate stabilization within safe limits. Hopes for CDR have largely been pinned on the extensive deployment of bioenergy with carbon capture and storage (BECCS), which is the only negative emissions technology included in the mitigation scenarios of the recent Intergovernmental Panel on Climate Change (IPCC) fifth Assessment Report^3^. BECCS achieves a net removal of CO_2_ from the atmosphere by relying on photosynthesis to fix CO_2_ and interrupting the natural carbon cycle that would otherwise have rapidly returned this carbon to the atmosphere during respiration or combustion. Instead, BECCS sequesters the photosynthetically fixed carbon as post-combustion CO_2_ stored in a stable reservoir^4^,^5^. However, BECCS may be costly and parts of the technology are unproven^6^.

An alternative CDR approach is biochar (BC), in which biomass is pyrolysed and the carbon-rich residue sequestered in soil. BC, like BECCS, relies on photosynthesis to fix CO_2_, followed by storage of this fixed carbon in a stable reservoir (in this case, soil). As a co-benefit, BC can improve the fertility of degraded or less fertile soils^7^,^8^,^9^. It is also possible to co-produce bioenergy with BC in a bioenergy-biochar system (BEBCS)^10^,^11^,^12^.

Within any mitigation scenario, BECCS and BEBCS must compete for available biomass—with each other and also with pure bioenergy systems (BES), which can also reduce energy-sector carbon emissions by offsetting more carbon intensive energy resources. Each of these technologies has advantages and disadvantages. BES generates the greatest bioenergy per unit biomass. BECCS sequesters the largest fraction of biomass carbon, but generates less energy than BES at a higher cost^13^,^14^. BEBCS produces the least energy and sequesters less carbon than BECCS^7^,^10^,^13^,^15^, but it can enhance soil fertility^16^,^17^, return nutrients to soil (although nitrogen may be largely lost or inaccessible to plants after pyrolysis), and reduce greenhouse gas (GHG) emissions from soils^16^,^18^. These BEBCS co-benefits potentially offset some of the cost of its CDR, and simultaneously address issues of food security for a growing world population^19^. BC can also increase or decrease the turnover rate of native soil organic carbon (SOC)^20^,^21^,^22^,^23^, which would create a negative or positive feedback, respectively, on the amount of carbon sequestered. BEBCS also has additional costs associated with haulage, handling and tillage of BC.

Under which, if any, set of biophysical and economic conditions BEBCS becomes more attractive relative to both BES and BECCS has not been previously investigated. We address this issue here by developing a multi-parameter model to determine which of BES, BECCS, or BEBCS is the more economically efficient use of a given biomass resource for climate-change mitigation under varying conditions. A multivariate Monte Carlo analysis was conducted using a broad range of parameter related to agro-ecological, economic, technological, global spatial and temporal variability and uncertainty to 2100. Here we show that, despite BECCS offering twice the carbon sequestration and bioenergy per unit biomass, BEBCS may allow earlier deployment of CDR at lower carbon prices. Our model predicts that, at carbon prices above $1,000 Mg^−1^ C, BECCS is typically the most economic biomass technology for climate-change mitigation. At carbon prices below $1,000 Mg^−1^ C, BEBCS is the most cost-effective technology only where BC significantly improves agricultural yields, with pure BES being otherwise preferred. Thus, we have shown that BEBCS can be competitive with both BES and BECCS under a range of conditions.

## Results

### Impact of carbon price on optimal technology

Biomass supply chains are highly heterogeneous, with costs and emissions varying geospatially and temporally with species, production conditions, and harvest, collection and storage practices. To facilitate comparison of biomass conversion technologies across this diverse landscape of feedstock supply chains, the analysis was based on a functional unit of mass of biomass delivered to the conversion facility gate, assuming that all costs and emissions associated with delivering that biomass are independent of the conversion technology used to process it. For each of the conversion technologies (BES, BECCS or BEBCS), we define its relative net present value (RPV) as the difference between its net present value (NPV) and the NPV of the best alternative. By making comparisons on the basis of RPV per unit biomass, all costs, benefits and emissions associated with biomass supply cancel out. For a given biomass supply chain, we thus calculate which of the technologies is most economically efficient relative to the alternatives under given conditions, with the caveat that this approach does not determine when biomass price or environmental externalities would make none of the bioenergy conversion processes viable. A full description of the calculation of RPV is provided in Methods.

**Figure 1:**
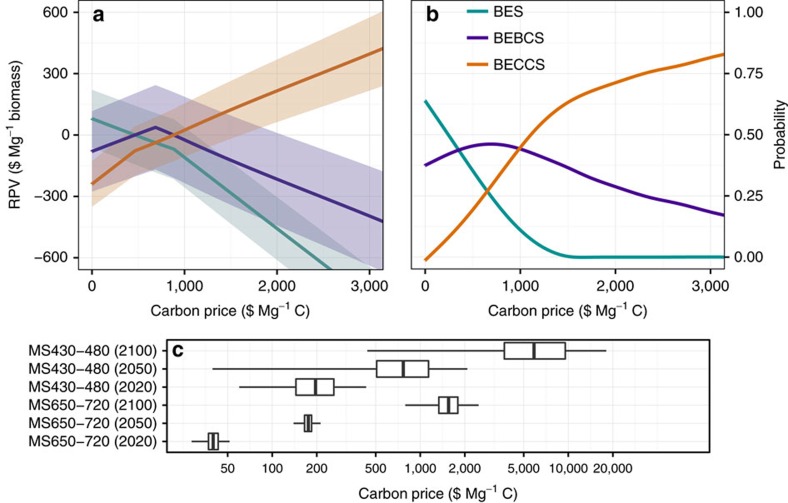
Relative present value as a function of carbon price. (**a**) Shows the relative present value (RPV) and (**b**) shows the probability of having the highest RPV, as a function of carbon price, for bioenergy systems (BES; cyan line), bioenergy with carbon capture and storage (BECCS; orange line) and bioenergy-biochar systems (BEBCS; purple line). Shading indicates one s.d.. (**c**) Shows the carbon price distribution for each of the mitigation scenarios (MS430–480 and MS650–720) in each of the years 2020, 2050 and 2100 (boxes indicate the median, upper and lower quartiles and whiskers extend to 1.5 times the interquartile range).

At carbon prices below $460 Mg^−1^ C, BES has the highest mean NPV (range where BES mean RPV>0 in Fig. 1a). For carbon prices in the range of $460–$900 Mg^−1^ C, BEBCS has the highest mean NPV. Above $900 Mg^−1^ C, BECCS has the highest mean NPV (Fig. 1a).

Although these three technologies can be broadly categorized by which is optimal at different carbon prices, variability (and, to a lesser extent, uncertainty) in their RPVs contributes to considerable overlap in the ranges of suitability of the three technologies (see overlapping uncertainty ranges shown by the shaded areas at +/− 1 s.d. (*σ*) in Fig. 1a). In the lower carbon-price range below $400 Mg^−1^ C, BES is most likely (*P*=0.44–0.64) to have the highest NPV, while BEBCS has the highest NPV for most other situations (*P*=0.37–0.44), BECCS being infrequently (*P*<0.1) the most economic biomass power generation technology in this carbon-price range (Fig. 1b). Although BECCS is typically the technology with highest NPV in the higher carbon-price range (>$1,000 Mg^−1^ C), even with carbon prices as high as $3,000 Mg^−1^ C BEBCs remains the most economic technology in 20% of the parameter space (Fig. 1b).

Two mitigation scenarios were investigated: MS430–480, in which atmospheric CO_2_ concentration in 2100 is in the range 430–480 p.p.m. (IPCC Representative Concentration Pathway RCP2.6), consistent with a likely (defined as *P*>0.66) probability of temperature change below 2 °C relative to pre-industrial levels; and MS650–720, which relates to the upper band of IPCC RCP4.5, in which temperature change below 2 °C is unlikely, but<3 °C warming remains more likely than not^3^ (*P*>0.5). The carbon price ranges for each of the mitigation scenarios (MS430–480 and MS650–720) in each of the years 2020, 2050 and 2100 are shown in Fig. 1c to facilitate cross referencing of the carbon prices in Fig. 1a,b to the scenarios (see Supplementary Fig. 1 for cumulative probability distributions of carbon prices in each scenario, and Supplementary Table 1 for summary statistics of these distributions).

### Relative importance of parameters

**Figure 2:**
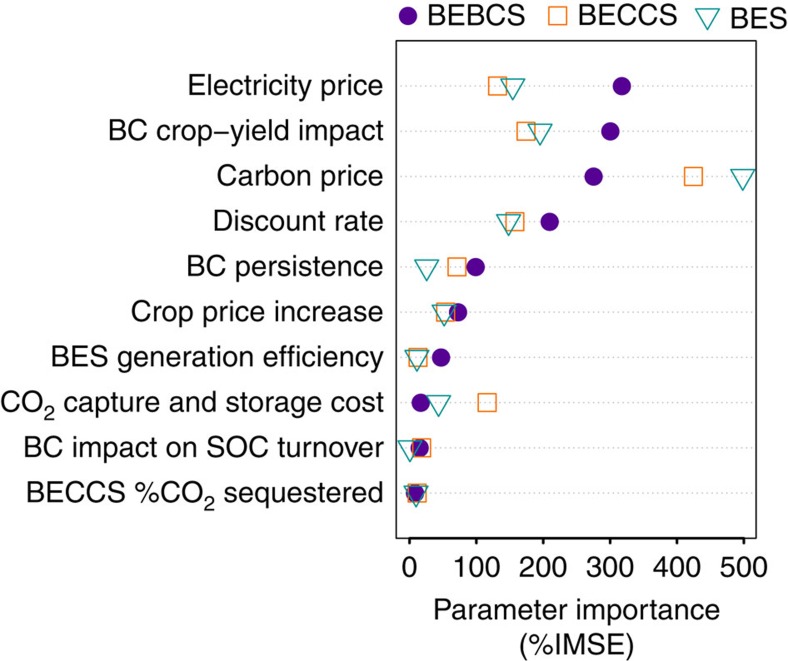
Parameter importance as determinants of relative present value. The most important parameters for determining the relative present value (RPV) of bioenergy systems (BES; open cyan triangles), bioenergy with carbon capture and storage (BECCS; open orange squares) and bioenergy-biochar systems (BEBCS; filled purple circles). Parameters are ranked in order of importance in determining BEBCS RPV. Parameter importance was determined by random forest analysis incremental mean square error (IMSE). BC, biochar; CCS, carbon capture and storage; SOC, soil organic carbon; and BECCS seq. fraction is the fraction of generated CO_2_ that is sequestered.

The relative importance of parameters in determining variability in RPV was assessed by random forest analysis, a method that takes account of parameter covariances^24^. Parameter importance was estimated by incremental mean square error (IMSE) of the random forest model with respect to each parameter (Fig. 2).

The highest ranked parameters, in order of their importance as determinants of BEBCS RPV are electricity price, BC’s long-term impact on crop yields, carbon price and discount rate. Electricity price and BC’s crop-yield impact are of approximately equal importance because the value of BC to crop production must offset the foregone energy production involved in its manufacture for BEBCS to be preferred. Note that it is the long-term impact of BC on alleviating soil constraints to crop yields that is critical to its economic value. BC is most likely to have a long-term positive impact on crop production in soils with low cation exchange capacity (CEC) (Methods: crop yield impacts of BC). While BC can also improve crop yields through ephemeral mechanisms such as fertilization (when BC contains plant-available nutrients) or raising the pH of acidic soils, these mechanisms have lower economic value, because their impact diminishes rapidly over time (Methods: value of BC’s liming potential to address soil pH constraints”, and “value of BC’s nutrient content). The next most important determinant of BEBCS RPV was the persistence or stability of BC in soil (Methods: BC decomposition, and Supplementary Fig. 2).

The ranking order of most important parameters differed slightly for BES and BECCS, in which cases carbon price ranked highest. Carbon price is more important for BES and BECCS, because it is the primary factor in determining which of these two is more cost effective, with low (high) carbon prices favouring BES (BECCS), with other factors altering the balance between them in the intermediate price range. BEBCS, on the other hand, is slightly less sensitive to carbon price than BES or BECCS, because it has an additional major value stream—its crop yield impact—that is independent of carbon pricing. Discount rate ranks highly because of its differential impact on the various technologies, with BC continuing to accrue benefits gradually over time after it has been produced (due to its impacts on soil fertility and soil GHG emissions), whereas the revenue streams from BES and BECCS (energy production and carbon sequestration) are valued at time of production.

**Figure 3:**
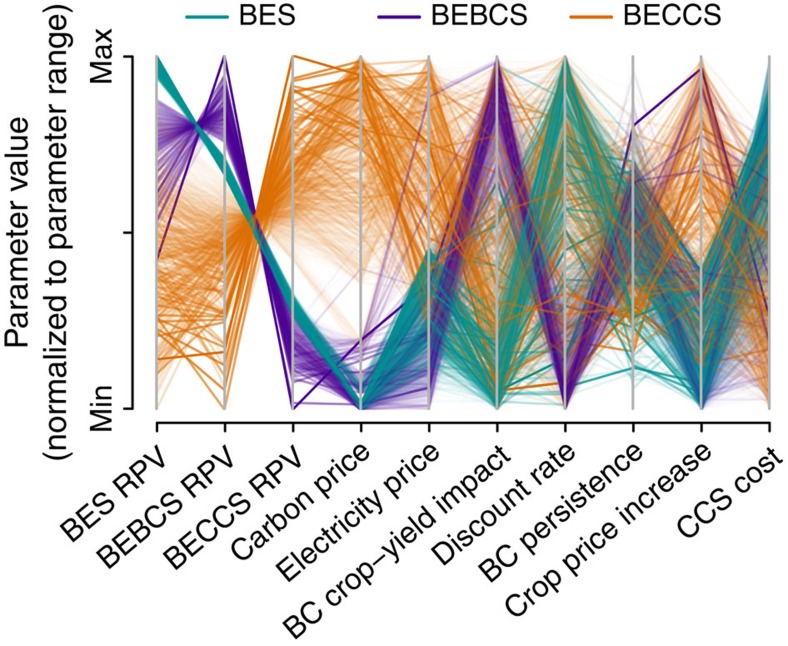
Parallel coordinates plot of the relative net present values as a function of the most important parameters. Relative net present values (RPVs) of bioenergy systems (BES), bioenergy with carbon capture and storage (BECCS) and bioenergy-biochar systems (BEBCS), and the principal factors on which these RPVs depend, in the years 2020, 2050 and 2100, are shown here with each of the seven most important parameters (see Fig. 2) plotted on parallel axes, to allow depiction of a multidimensional parameter space in a two-dimensional image. Each line represents a single data point in the Monte Carlo simulation, connecting the values of each variable for that point. The number of Monte Carlo simulations plotted was limited to 4,500 (750 randomly sampled from each of the six scenario-year combinations) to prevent over-cluttering the plot and to ensure high visibility of the main trends. Parameter values are normalized linearly within their sample ranges so that the *y* axes of all parameters share a common scale that is linearly interpolated between the parameter minimum and maximum. Line transparency is graduated by RPV (darker, less transparent lines for higher RPV), also to increase visibility of the main trends. Lines are coloured by which technology is optimal under the specified conditions (cyan, BES, purple, BEBCS and orange, BECCS).

Plotting the RPVs and the highest-ranked parameters on a parallel coordinates plot (Fig. 3) provides a useful visual indication of the typical conditions in which the RPV of each technology is maximized. Each line on the parallel coordinates plot corresponds to a single point in the multi-dimensional parameter space, with lines connecting the values for each parameter on their respective (parallel) axes. Each parallel parameter axis is normalized to range from the minimum to the maximum value of that parameter. For example, it can be seen that a high RPV for BECCS (Fig. 3, orange lines) correlates with high carbon and electricity prices (evident in the clustering of orange lines at the upper end on these parameter axes), whereas the opposite conditions favour BEBCS. When interpreting such plots, however, care must be taken to consider covariance between parameters, which can give rise to correlations that are not causal. For example, it can be seen in Fig. 3 that large increases in crop price correlate with a high RPV for BECCS, despite the fact that high crop prices would favour BEBCS rather than BECCS if all else remained equal. But, the time factor in the model means that crop prices rise over time (Methods: future food price trends), while other factors that improve the competitiveness of BECCS also increase with time and thus co-vary with crop prices without any causal link.

### Impacts of mitigation scenario and time on technology mix

**Figure 4:**
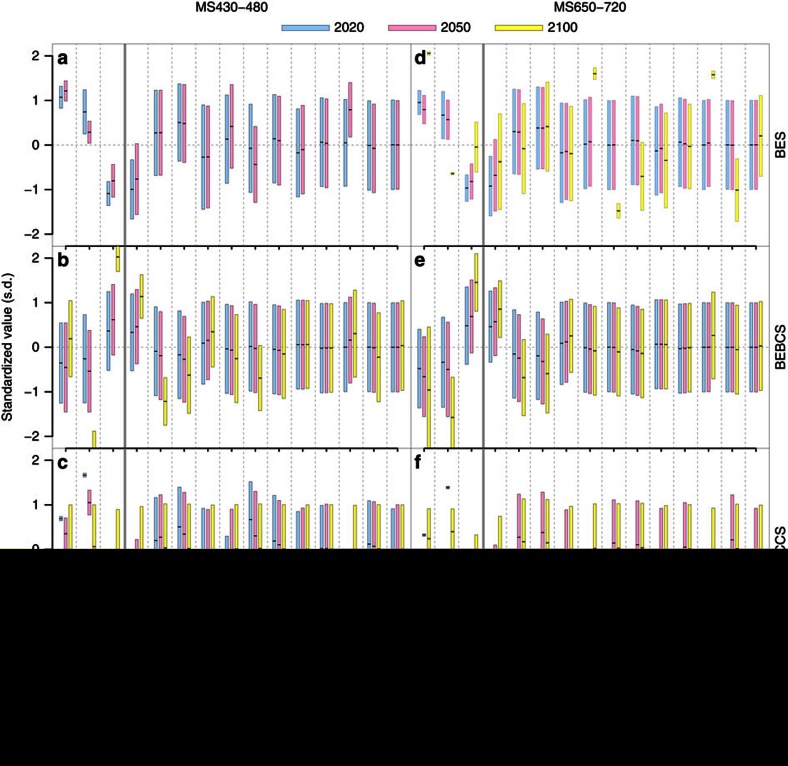
Parameter ranges for which each technology has the highest relative net present value. The *y* axes are in units of standardized values for each parameter—that is, population mean of that parameter (in each scenario) is at zero, and height is measured in s.d.’s from the mean. (**a**–**c**) Relate to mitigation scenario MS430–480 (that is, atmospheric CO_2_ concentration in 2100 is 430–480 p.p.m. (IPCC Representative Concentration Pathway RCP2.6), consistent with a likely chance to keep temperature change below 2 °C relative to pre-industrial levels), and **d**–**f** relate to MS650–720 (RCP 4.5, in which temperature change below 3 °C is more unlikely than likely). Each panel is divided in two with three response variables (relative present values of BES, BEBCS or BECCS) on the left of the solid line, and the 12 most important independent parameters on the right. Each parameter has three bars (blue, pink and yellow) for parameter ranges in the years 2020, 2050 and 2100, respectively. Bars show the mean +/− 1 s.d. of the samples in which BES (**a**,**d**), BEBCS (**b**,**e**) or BECCS (**c**,**f**) has the highest RPV. Thus, the graph shows for each scenario (level of mitigation and year) how far each parameter must differ from the mean of that parameter in order to make each technology option the preferred one. Note that absent bars for BECCS in 2020 (**f**) and BES in 2100 (**a**) indicate that they are not economically viable at those times.

A more detailed approach to quantifying and visualizing the impacts and interactions of parameters was investigated using the distributions of parameter values for which BES, BEBCS or BECCS becomes the preferred technology, disaggregated by mitigation scenario and time (Fig. 4). Figure 4 indicates for each scenario (level of mitigation and year) how far each parameter must differ from the mean of that parameter in order to make each technology option the optimal one. In the following discussion, the bias, *δ*, of parameter *P* is defined as *δ*=[*μ*(*P,T*)−*μ*(*P*)]/*σ*(*P*), where *μ*(*P*) and *σ*(*P*) are the mean and s.d., respectively, of parameter *P*, and *μ*(*P,T*) is the mean when technology *T*∈{BES, BEBCS, BECCS} has the highest RPV. That is, *δ* provides a measure (in dimensionless units of s.d.’s) of how far, on average, values of *P* must deviate from the mean for a given technology to be most economic. A high value of *δ* indicates that a parameter exerts a strong effect on whether a technology will be the preferred option, with positive (negative) values of *δ* indicating that the parameter must be higher (lower) than average to make that technology optimal. Absolute values of the ranges, means and s.d.’s of each parameter in each scenario are given in Supplementary Tables 2–4.

In 2020–2050, BES is the preferred technology option when its RPV is, on average, 0.8–1.2 s.d.’s higher than its mean value (that is, *δ*=0.8–1.2). For BES to achieve this higher than average RPV requires conditions that favour BES relative to BEBCS (the primary competing technology at the carbon prices prevailing in 2020–2050 even under the more stringent mitigation scenario MS430–480). The main factors that favour BES over BEBCS are: a lower than average BC yield impact (*δ*=−0.7 to −1.0 *σ*), higher than average energy prices (+0.4 to +0.5 *σ*), and low discount rates (−0.3 *σ*). By 2050 in MS430–480, lower than average carbon prices (−0.4 *σ*) (as carbon prices start to move into a range where BECCS is optimal under favourable conditions) and above average carbon intensity of the electricity generation sector (+0.4 *σ*) also begin to have a stronger role in determining if BES will be the preferred technology.

By 2100, BES is not competitive in MS430–480 under any conditions (note the absence of bars for BES in this scenario in 2100 in Fig. 4a). In MS650–720, on the other hand, BES maintains its competitiveness in 2100 only when a combination of factors are all seen, including a high carbon intensity (+1.6 *σ*), low carbon prices (−1.5 *σ*), high cost of carbon capture and storage (+1.6 *σ*), and a low fraction of CO_2_ being sequestered by BECCS (−1.0 *σ*).

In 2020–2050, BEBCS is optimal under roughly the opposite conditions that favour BES (BES and BEBCS being the main competing technologies at these times). In essence, this means that BEBCS is most likely to be favoured when the BC yield impact is higher than average (+0.4 to +0.6 *σ*), energy prices are below average (−0.2 to −0.4 *σ*), and discount rates are slightly below average (− 0.2 *σ*). By 2100 in MS650–720, more stringent conditions are required to keep BEBCS optimal, with a higher yield impact (+0.9 *σ*), lower discount rate (−0.7 *σ*), and lower electricity prices (−0.6 *σ*) being required. Whereas, in MS430–480, BEBCS remains competitive by 2100 with BECCS only under particularly favourable conditions that give BEBCS an RPV +2.0 *σ* higher than average. These conditions depend on a combination of a very low discount rate (−1.2 *σ*), a carbon price that is in the lower range for that time (−0.7 *σ*), and locations with soils that show a high BC yield impact (+1.1 *σ*), which is most likely to occur on cropland soils with low CEC (Methods: crop yields impact of BC).

BECCS is not competitive in 2020 in MS650–720 under any conditions (hence, the bars for BECCS are absent for these years in Fig. 4f). In 2020, BECCS is only competitive in MS430–480 under highly favourable conditions that require a very low cost for carbon dioxide capture and sequestration (CCS) (−1.6 *σ*), low BC yield impact (−1.0 *σ*), and high carbon prices (+0.7 *σ*). For BECCS to be competitive in 2050 in the more ambitious mitigation scenario MS430–480 requires that a combination of factors improve its performance relative to both BES and BEBCS simultaneously, both of which are also viable technologies under a wide range of conditions at that time. This requires some combination of low BC yield impact (−0.6 *σ*), high carbon price (+0.3 *σ*), low CCS cost (−0.8 *σ*) and high discount rate (+0.3 *σ*). By 2100 in MS430–480, BECCS is favoured under most conditions, therefore, no significant bias is observed in any of the parameters to make BECCS competitive. The same is broadly true in MS650–720, except that BEBCS can remain competitive in 2100 when its yield impact is high (+0.9 *σ*), the discount rate is low (−0.7 *σ*), and electricity prices are low (−0.6 *σ*).

## Discussion

Our results show that the availability and inclusion of BEBCS within a portfolio of climate-mitigation measures can reduce the costs and ease the implementation of long-term CDR strategies using biomass, because BEBCS can be significantly cheaper than BECCS when agronomic benefits offset costs. The reduced cost of CDR using BEBCS, relative to an energy supply portfolio in which BECCS is the only negative emissions technology, may allow for earlier CDR deployment at a lower carbon price than in scenarios without BEBCS.

However, long-term improvements in soil productivity from BC applications are required for BEBCS to become competitive with either BES or BECCS. This suggests that an integrated portfolio of BECCS plus BEBCS would maximize the economic potential for CDR, with BEBCS being preferred where long-term fertility of agricultural soils can benefit most from BC. However, it is important to note that long-term crop trials with BC remain largely absent from the published literature. Of the 781 treatments from 68 published studies used to develop the crop response relationship for our model, 85% were measured in the first year following BC addition, 10% in the second year, 3% in the third year, 2% in the fourth year and no data were available from longer term trials in the peer-reviewed literature (Methods: Crop yield impacts of BC, Supplementary Figs 3–5, and Supplementary Tables 5 and 6). Although a correlation between higher crop response and low soil CEC was found, suggesting that BC can offer long term improvement to the fertility of low CEC soils (Methods: Crop yield impacts of BC), the evidence to support this hypothesis from direct observation remains inadequate. Furthermore, in some studies, unfavourable changes in soil chemical, physical and biological properties and reductions in crop yields have been reported^25^. An improved understanding of the mechanisms underlying crop response to BC will support the development of decision-support models that direct BC applications into systems they can most benefit, and that avoid the application of unsuitable grades of BC in cropping systems where they may do harm, while distinguishing between uncertainty and manageable variability^26^. Nonetheless, we note that there is currently low confidence and high uncertainty in the expected long-term response of specific soil-crop systems to BC amendments. While the parameter ranges used in our model reflect this high level of uncertainty, it is clear that further research to provide long term data on crop response in a variety of soils, cropping systems and agroecological zones should be a pre-requisite to the widespread use of BC. Further research will also be required to quantify impacts on ecosystem services and functions such as interactions with soil-dwelling and aquatic biota, nutrient cycling through ecosystems and the potential for contamination or bioaccumulation of contaminants^27^.

Where or when BEBCS is not viable, BES (BECCS) is more often than not (that is, *P*>0.5) the preferred option at carbon prices below (above) $660 Mg^−1^ C. The dependence of BEBCS on beneficial soil-fertility impacts to be economically viable means that its economic potential for climate-change mitigation is likely lower than its sustainable technical potential previously reported^7^. BEBCS may, however, increase the total technical potential for CDR, not just because it allows for earlier deployment, but also because it may be applicable in situations where BECCS is not: for example, in locations too remote from suitable geologic or other sinks for BECCS to be economic, or in the production of carbon-negative transport biofuels—a sector not readily compatible with CO_2_ capture and storage. The results reported here indicate that BEBCS could be a key component to reduce the costs and otherwise ease the implementation of long-term climate-mitigation strategies.

At present, integrated assessment models (IAMs) are the primary tool for assessing questions such as these associated with long-term stabilization scenarios. While recognizing that it is premature to embark on a large scale deployment of BEBCS for climate-change mitigation, given the remaining uncertainties outlined above, we recommend that a useful next phase of research would be inclusion of BEBCS as a technology option in IAMs. Large uncertainties are routinely investigated in IAMs by the use of scenarios as a means to provide what-if analyses. For example, the current uncertainty over the viability of CCS as a mitigation technology option, has been investigated in IAM simulations through the use of scenarios in which CCS either is or is not included as an option (see, for example, fig. 6.35 in ref. 4). Including scenarios, both with and without BEBCS, in IAM simulations would allow for a more thorough understanding of how availability of BEBCS as a mitigation option would affect the cost, timing and technology mix of a comprehensive mitigation strategy, if increased crop yields persist in the long term. This improved understanding will be critical to making informed decisions about how great an investment in further BC research is justified. For example, we have shown that, with carbon prices expected to rise over time, BEBCS could lead to earlier adoption of CDR. However, the converse is also possible, that BEBCS might delay adoption of BECCS until carbon prices rise sufficiently high for BECCS to become competitive with BEBCS. The overall impact of such interactions on overall mitigation outcomes and costs can only be assessed adequately by using IAMs.

A final issue is the question of the costs and impacts of biomass supply chains to support any of these technologies at a sufficient scale to make a substantial contribution to climate change mitigation. The land area required to provide sufficient BECCS to meet the requirements of MS430–480 has been estimated at 400–700 Mha (two to four times the global area of abandoned or marginal land)^28^. These large demands on land would be expected to create competition for productive land with food, fibre, biofuels, habitat and other ecosystem services^29^,^30^. Nutrient^28^,^31^ and water^32^ supply may also further constrain biomass supply potential. The results presented here are independent of the actual costs (economic, social and environmental) and prices of biomass supply, because we calculate only the relative value of each technology (that is, how much better or worse it is than the alternative technologies considered). In practice, these costs and the price of biomass will be key determinants of the feasibility of any mitigation strategy that places demands on biomass supply. At present, there is low agreement in the literature as to the scale of the biomass potential once economic, social and environmental impacts are considered. Improved estimates using a systems approach that considers all these aspects in a spatially explicit manner must be a high research priority to ensure that policy decisions with possibly irreversible global implications are not based on unrealistic estimates of the biomass supply potential.

## Methods

### Overall approach

A discounted cost-benefit analysis approach was used to compare BES, BECCS and BEBCS, with global environmental benefits of avoided carbon dioxide emissions internalized into the econometric model as a carbon price. NPV is defined as the sum of all time-discounted costs and benefits of a project (equation (1))


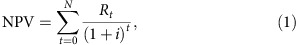


where, *t*=time interval, *N* is the total number of time intervals over the period of analysis, *R*_*t*_ is the net cash flow (total benefits minus total costs) at time *t*, and *i* is the discount rate.

Comparative evaluation of the three biomass conversion technologies (BES, BECCS and BEBCS) was conducted by calculating how much higher or lower each technology’s NPV is than the next best alternative—a metric we refer to as its RPV, defined by equation (1):


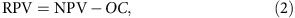


where OC is the NPV of the best alternative foregone. To calculate RPV for each technology, we note that by defining NPV’ as the NPV excluding costs and benefits associated with the biomass supply chain (NPV_bm_), the problem becomes more tractable by cancelling NPV_bm_ from the calculation (equations (3 and 4)):


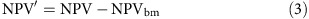


Therefore,


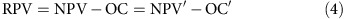


Hence, by making comparisons on the basis of a functional unit of mass of biomass delivered to the conversion facility gate, all costs, benefits and emissions associated with the biomass supply side of the equation cancel out and we are able to calculate, for a given biomass supply chain, which of the conversion technologies considered is most economically efficient.

The NPV calculation of the various conversion technologies consisted of the following components. First, all technologies have capital, operation and maintenance costs; revenue from energy production; and avoided CO_2_ emissions from reference energy system that would have provided energy supply if bioenergy were not generated. Second, BECCS and BEBCS have an additional benefit from reduced atmospheric CO_2_ due to their carbon sequestration component (that is, they return a smaller fraction of the biomass carbon to the atmosphere than do BES), but with lower energy conversion efficiencies than BES. Third, BEBCS can have further benefits from its potential to increase agricultural productivity on low-fertility soils, to reduce N_2_O emissions from soils, and to offset agricultural lime and fertilizer inputs. BEBCS has additional costs and emissions associated with transport of BC and field operations to spread and incorporate the BC. BC can also alter (increase or decrease) the turnover rate of native (non-pyrogenic) SOC, which can either be a cost or a benefit, depending on the direction of the effect.

Thus,


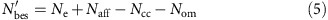



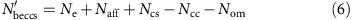



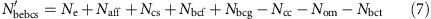


Where,

*N*_e_=NPV of net energy generated,

*N*_aff_=NPV of avoided fossil fuel emissions,

*N*_cc_=NPV of capital costs,

*N*_om_=NPV of operation and maintenance,

*N*_cs_=NPV of carbon sequestration,

*N*_bcf_=NPV of BC impacts on soil fertility and crop production

*N*_bcg_=NPV of BC impacts on soil GHG fluxes

*N*_bct_=NPV of BC transport and tillage operations

A Monte Carlo analysis was conducted (*n*=30,000 per scenario) to investigate the performance of each technology over a broad range of parameter values representative of uncertainty and variability globally to 2100. Ranges, means and s.d.’s of each parameter in each scenario are given in Supplementary Tables 2–4. Derivation of these parameter ranges and methods and equations used to model their impacts are detailed in the following sections.

### Carbon price

We apply a price on carbon to internalize impacts on net GHG fluxes into the economic comparison of biomass energy technologies. It was assumed that the same carbon price would apply to all technologies equally. The ranges of estimated carbon price within all idealized implementation scenarios that reported carbon prices in the IPCC WG III AR5 Scenario Database (Annex II.10) (ref. 33) are shown in Supplementary Table 1, categorized by year and by maximum atmospheric CO_2_ eq. concentration in 2100. The quantiles in Supplementary Table 1 for a given year and concentration pathway were fitted to lognormal distributions for multivariate sensitivity analysis; cumulative probability curves of these fitted functions are shown in Supplementary Fig. 1. Non-CO_2_ GHGs were converted to CO_2_ equivalents using 100-year global warming potentials^34^. Thus, each IPCC emission pathway was represented in the scenarios by the corresponding carbon price range (Supplementary Tables 1, 3 and 4). According to standard practice in integrated assessment modelling, the carbon price was applied both to net avoided emissions (for example, avoided fossil fuel consumption displaced by bioenergy) and also to carbon offsets in the form of net sequestered carbon in BECCS and BEBCS.

### Discount rate

There has been much debate in the recent literature about the appropriate discount rate to apply for issues with long-term and intergenerational impacts such as climate change. Discounting of future costs and benefits is done for two principal reasons. First, if the overall economy grows, as is assumed in many (but not all) forecasts, the utility of a given sum of money diminishes according to the law of diminishing marginal utility. Second, and more contentiously, future costs and benefits are often discounted simply because they occur further in the future. This second component of discounting is referred to as the pure time discount rate (PTDR) and is a common feature of most markets. The Stern review^35^ challenged the prevailing application of a PTDR, arguing that it is indefensible with regard to inter-generational ethics. For example, Stern *et al*. (page 31 of ref. 35) state, ‘we take a simple approach in this review: if a future generation will be present, we suppose that it has the same claim on our ethical attention as the current one’. This stance has been challenged by others who argue that applying a PTDR is more consistent with observed market behaviour^36^, and with increasing uncertainty associated with forecasting the distant future^37^. We do not take a specific stance here on the correct social discount rate to apply, but use a sensitivity range in the Monte Carlo analysis that covers the range of values normally applied as social discount rates, from the lower value of 1.5% per year suggested by Stern *et al*.^35^, to the upper bound of 6% suggested by both Nordhaus^36^ and Weitzman^37^.

It should be noted that BES is, in comparison to BECCS and BEBCS a more mature technology. Therefore, it is currently considered to be less risky to invest in this technology. Risk is sometimes reflected in discount rates, with higher (risk-adjusted) discount rates being applied to higher risk technologies. In this analysis we account for risk, not by applying differential discount rates to each technology, but by using a Monte Carlo method to simulate uncertainty in a technology performing worse (or better) than expected. Expected cash flows are thus adjusted in line with the probability distributions of expected returns. We have thus used the conventional practice of applying a common social discount rate to quantify the uncertainty in the net social costs and benefits for each technology on a common basis. It should be borne in mind, however, that risk-averse investors may, in practice, discount less proven technologies at a higher rate.

### Capital depreciation

Capital expenditures were depreciated at the discount rate (*i*) over a plant lifetime (*L*) within the sensitivity range of 25–45 years. Annualized cost of capital was thus calculated by equations (8 and 9) below.


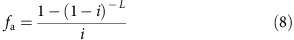



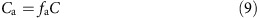


Where, *C* is the total capital cost, *C*_a_ is the annualized capital cost and *f*_a_ is the annuity factor.

### Leakage and macroeconomic effects

Here we define leakage in the sense defined by the IPCC as a change in GHG emissions occurring outside of the project boundary resulting from project activities. This study investigates the marginal NPV of adding a unit of biomass power generation in the form of BES, BECCS or BEBCS. As such, it assumes that the overall economic system is not significantly perturbed by the marginal change. Therefore, leakage arising from broader economic effects due to changes in energy, carbon, food, land or other prices as a result of adding bioenergy capacity were beyond the scope of this study.

### Reference energy system

It was assumed that electricity provided by the BES (BES, BEBCS or BECCS) would otherwise have been provided by a reference energy system with carbon intensity CI_ref_. In practice CI_ref_ depends what type of power generation technology would have supplied the energy in the absence of the bioenergy production. Depending on the legislative and economic framework within which the BES is implemented, there can be several different types of drivers of this interaction. Factors that can influence the carbon intensity of offset energy include financial and legislative instruments that may be designed to ensure that new bioenergy capacity allows phase out of the most polluting alternative power sources. Also, competition for available mitigation incentives can lead to bioenergy capacity offsetting other low-carbon alternatives that would otherwise have received available finance. Additionally, market forces or planning objectives may lead to a mixed effect in which bioenergy offsets a mix of technologies whose combined carbon intensity could be the overall energy mix of the existing system, the energy mix of a subset of the existing energy infrastructure (for example, base load or load-following power generation), or the energy mix of new plant that would otherwise have replaced existing capacity as it reaches end-of-life.

To account for all these possibilities, we use a wide sensitivity range for CI_ref_ in all scenarios that includes both high- and low-carbon technologies (Supplementary Table 7) as a Gaussian distribution with mean and s.d. as given in Supplementary Table 8. In 2010, the global carbon intensity of electricity generation was 0.04 Mg C GJ_e_^−1^(where GJ_e_ is a GJ of electrical energy generated (page 741, fig.7.7 in ref. 38). Avoided carbon emissions from the reference energy system (AE_ref_) were then calculated as electricity produced by BES (*E*_bes_) multiplied by carbon intensity of the reference energy system (CI_ref_) (equation (9)):


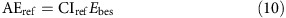


### Electricity price

Mean electricity price over 2010 to 2013 in IEA countries was US$ 32.78 +/− 9.67 GJ^−1^ (1 s.d.) (equivalent to 11.8 +/− 3.5 c kWh^−1^), industrial price before tax^39^. The lowest price was 14.59 USD GJ^−1^ (Norway), and the highest was 56.32 USD GJ^−1^ (Italy). However, power generation in Norway is currently over 90% hydro-electricity and may, therefore, be unrepresentative of prices for biomass energy. The next lowest price was in the USA, at 17.88 USD GJ^−1^, which was therefore used as the lower bound of the range. In all, 2014 electricity prices were, accordingly, assumed to lay in the range 14.6–56.3 USD GJ^−1^. Electricity prices to 2100 were estimated to rise linearly at 1.9–2.1 % of 2014 values per year^40^.

### Bioenergy systems

Capital costs, generation efficiencies and operation and maintenance costs for biomass power generation are shown in Supplementary Table 9, for both recent (2012) plant, and for projections to 2030. It was assumed that there would be no significant further cost reductions or efficiency improvements beyond the 2030 values. Values in 2020 were estimated by linear interpolation. To provide comparability with BECCS, which is typically only considered plausible at the large scale, the parameter ranges for >50 MW plant were used in the Monte Carlo sensitivity analysis.

### Bioenergy with carbon capture and storage

The baseline capital, operation and maintenance costs of the biomass energy component of BECCS were assumed to be the same as for BES, but with additional costs associated with CCS as described in the following section.

### Cost of carbon capture and storage

Early IPCC estimates of the capital cost for CCS applied to coal power stations were in the range $12–52 Mg^−1^ CO_2_ captured (2007 basis, with lower costs being associated with integrated gasification-combined cycle, and higher costs with pulverized coal plants)^15^,^41^. Later estimates based on greater experience and closer examination estimate tended towards higher CO_2_ capture costs in the range of $48–80 Mg^−1^ CO_2_ captured ($67–109 Mg^−1^ CO_2_ avoided)^42^,^43^,^44^,^45^. The most recent and detailed estimates, which we use for this study, expand this range somewhat to between $28–111 Mg^−1^ CO_2_ captured for CCS applied to fossil fuel power plants^46^. These estimates, however, assume a capacity factor of up to 0.85 which is high over the lifetime of a plant when compared with recent experience of base-load coal power stations which on average have achieved levelled capacity factors of 0.65–0.75 in the USA^47^. Here we assume a capacity factor in the range 0.65–0.85, potentially increasing fixed capital costs by up to 24% relative to estimates based on a capacity factor of 0.85. We apply the same capacity factor for BECCS, BEBCS and BES to prevent bias.

Costs of transporting CO_2_ were estimated to range from $1.3 to 14.8 Mg^−1^ CO_2_ per 250 km and geological storage costs (including monitoring) to range from $0.6–8.3 Mg^−1^ CO_2_ (ref. 41). The pipeline diameter required for CO_2_ transport increases more slowly with flow rate (approximately with the square root of flow rate) than does cost per unit length (approximately proportional to diameter), giving rise to substantial economies of scale^48^. Collection of CO_2_ from a network of smaller biomass facilities co-located near to biomass resources would be likely to also entail longer transport distances than fossil fuel CCS. Although lower economies of scale and less efficient source-sink matching for BECCS relative to fossil CCS would potentially raise CO_2_ transport costs by up to $30 Mg^−1^ CO_2_ when CO_2_ must be transported further than 500 km (ref. 43), we assumed here that BECCS would not be applied in situations where biomass resources were too distant from suitable sinks to be viable^14^. CO_2_ transport distance was assumed to range from 1 to 500 km.

CCS applied to biomass power, where specifically considered in the studies cited above, has been assumed to have the same or comparable capital cost per kW of plant generating capacity as coal-based systems^43^. Biomass conversion facilities are likely to be smaller than fossil fuel power generation and would, therefore, tend to accrue lower economies of scale, with accordingly higher CCS costs, on average, per unit C captured than coal-based CCS^43^. We, therefore, extend the upper end of the range of CCS costs considered here to include also BECCS deployment in suboptimal conditions with lower economies of scale (up to 20% higher capital cost assumed), lower capacity factors and longer CO_2_ transport distance than coal-CCS (Supplementary Table 10). The costs shown in Supplementary Table 10 thus represents a range from: (a) biomass co-firing in large scale coal-powered power plant situated close to geological sequestration reservoirs, at the low end, to (b) medium scale dedicated BECCS (with 20% higher unit capital cost for CCS than a large scale facility), with a capacity factor of 0.65, located up to 500 km from reservoirs.

### Outlook for future CCS costs

Cost reductions of ∼15% have been estimated for combustion-based plants with CCS and 20% for gasification-based plants with CCS after 100 GW of increased capacity worldwide relative to current technology^46^,^49^. Accordingly, we have assumed cost reductions of 20% for CCS in 2100, interpolated linearly for 2050 (Supplementary Tables 3 and 4).

### BECCS efficiency penalty

Net generating efficiency, on a lower heating value basis is assumed to be lower with than without CCS by an efficiency penalty (*P*) of 4–11 percentage points^14^,^15^,^41^,^43^ (equation (11)):


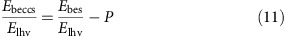


Where, *E*_beccs_ and *E*_bes_ are the net amounts of electricity exported from a BECCS and BES plant, respectively; and *E*_lhv_ is the energy content (on an lower heating value basis) of the biomass used to generate that electricity.

### BECCS CO_2_ sequestration fraction

We assume that between 81–91 % of CO_2_ generated from biomass combustion is sequestered^41^. Note that, per unit electricity generated, CO_2_ captured is greater than CO_2_ avoided, because the efficiency penalty (see BECCS efficiency penalty, above) means that more CO_2_ is emitted per GJ electricity. Care must be taken when comparing literature values as to whether they refer to CO_2_ captured or avoided. Here we base calculations on CO_2_ captured per unit biomass combusted.

### BEBCS costs

BC production necessarily entails a reduction in possible energy production per unit biomass than a pure BES, because a substantial fraction of the biomass enthalpy remains embodied in the BC product. Different pyrolysis technologies have different relative yields of energy and BC; fast pyrolysis producing more energy and less BC than slow pyrolysis. When energy is the desired product, BC produced during pyrolysis will typically itself be combusted or gasified to raise the energy production efficiency of the overall process. However, under conditions when it is economically viable to produce BC at the expense of energy (that is, when the value of the BC for soil improvement and carbon sequestration is high enough to offset foregone energy when it is buried in soil), it would then be most economical to maximize BC yields. Therefore, the BC production technology we consider here is slow pyrolysis. We assume that fast pyrolysis would be the preferred technology over slow pyrolysis only in those conditions when energy is more valuable than BC, in which case the BC product of fast pyrolysis would itself be combusted rather than added to soil.

Techno-economic assessments of pyrolysis for bioenergy have typically focused more on fast pyrolysis (with its higher energy production potential) rather than slow pyrolysis^50^,^51^,^52^. Accordingly, there are few detailed estimates of capital costs of slow pyrolysis power generation plants in the published literature. To estimate a realistic uncertainty range for slow pyrolysis power generation capital costs, we, therefore, base the sensitivity range on costs for biomass integrated gasification combined cycle (BIGCC) power generation, for which a substantial literature exists. In an analogous pyrolysis-based configuration (PyGCC), the gasification stage would be replaced with a pyrolysis unit, a cyclone for char separation, and possibly an additional gas conditioning plant to accommodate the higher tar content of pyrolysis gases than gasifier syngas. Due to the lower energy conversion efficiency of slow pyrolysis than gasification, the pyrolysis unit would need to be sized larger per unit electricity generation than a BIGCC gasifier (or conversely, for a given biomass throughput, the electricity generation stage would be smaller in PyGCC than BIGCC). This will also engender an increased size in biomass handling costs per unit power output.

Gasification accounts for ∼25–30% of BIGCC capital cost^53^. Biomass handling and storage equipment accounts for a further 15–20% of BIGCC capital cost^53^,^54^. We assume that biomass handling and gasification costs are scaled from BIGCC to PyGCC in proportion to the ratio of syngas fuel produced per unit biomass in a gasifier or pyrolyser (on an energy basis). Energy conversion efficiency is in the range of 75–85% for gasification^55^,^56^. For pyrolysis, energy conversion efficiency is in the range 37–60% (see pyrolysis mass and energy balance, below), where higher values correspond to pyrolysis at 600 °C with biomass being used as the fuel for heat supply; and lower values correspond to pyrolysis at 450 °C, with syngas providing the fuel for heat supply). To account for additional costs of BC handling and tar cracking, we also add a further 25% of the pyrolyser cost to the overall PyGCC system^54^. Thus, we estimate that capital cost of gasification and biomass handling for PyGCC is in the range of 20–60% higher than BIGCC per unit electricity output capacity.

Operation and maintenance costs were assumed to be 3–5 % of capital costs, as for BES (Supplementary Table 9).

### Crop yield impacts of BC

BC can improve crop yields by a variety of mechanisms, including (a) direct provision of nutrients, (b) alleviating pH constraints, (c) improving fertilizer use efficiency (and thus nutrient uptake for a given fertilizer application rate) by increasing the soil CEC and (d) improving soil water holding capacity (WHC) (in light or sandy soils) or drainage (in clayey soils)^16^,^57^,^58^. Of these mechanisms, nutrient content and liming potential of BC are likely to be transient effects, whereas CEC and WHC impacts are associated with the carbon matrix provided by BC and are likely to at least persist, if not increase, while the BC remains. We, therefore, treat transient and persistent impacts of BC separately in the valuation of its yield impacts.


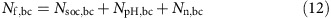


Where,

*N*_soc,bc_ is the NPV of BC’s long-term soil fertility benefits from increased SOC,

*N*_pH,bc_ is the NPV of BC’s liming potential and

*N*_n,bc_ is the NPV of BC’s nutrient content

To estimate the relative importance of these different types of mechanisms, we conducted a random forest decision-tree analysis^24^ of a comprehensive database crop trials with 781 treatments involving lignocellulosic feedstocks (excluding manures) from 68 published studies (Supplementary Fig. 3). The analysis was constrained to include only non-manure lignocellulosic feedstocks, because we are interested here in feedstocks that may have a competitive use in bioenergy power generation. Studies using hydrochar were also excluded because hydrochar turns over rapidly (thus having little sequestration potential), and its production is not currently compatible with power co-generation. Previous meta-analyses of published yield responses have not employed methods that account for both correlation (non-independence) and interactive effects of explanatory parameters^16^,^17^. The random forest approach does not have these limitations, and furthermore makes no implicit assumptions about the linearity or form of the model. The stochastic ensemble approach of random forests also eliminates the tendency of single decision tree models to overfit data^24^.

Of the BC yield-impact data shown in Supplementary Fig. 3, 85% were measured in the first year following BC addition, 10% in the second year, 3% in the third year, 2% in the fourth year and no data were available from longer term trials. Random forest analysis was conducted using the cForest function in the party package of the R statistical programming language, which provides random forest, bootstrapping and bagging ensemble algorithms utilizing conditional inference trees. cForest was run with an ensemble of 5,001 trees (parameter value ntree=5,001), 5 parameter tries per node (mtry=5), and the cforest_unbiased method to eliminate bias towards predictors with continuous values or greater number of categories. The resulting relative importance of the predictor variables (estimated by incremental change in mean square error from omitting a variable from the model) is shown in Supplementary Fig. 4.

The relative importance of the model parameters was estimated by the IMSE for each parameter in the random forest analysis^24^. Soil pH and CEC were overwhelmingly, and approximately equally, the most important predictors of yield response to BC (IMSE=0.085 and 0.084, respectively). Next, clay and silt content, BC carbon fraction, and fertilizer and BC application rates were of secondary importance (IMSE=0.02–0.04). Crop type, pyrolysis temperature, sand, SOC, BC pH and feedstock were only minor predictors of yield response (IMSE<0.02). Time since BC application was found to be a very poor indicator of yield response within the available data, but this is likely to have been skewed by most of the measurements being taken during the first cropping cycle following application.

### Value of increased soil carbon from BC additions

Because pH impacts of BC are approximately equally responsible for observed yield responses as are CEC effects (Supplementary Fig. 4), it is unlikely that the more extreme yield responses observed would be solely attributable to increased SOC, rather than having also a pH and possibly nutrient mechanism contributing. We, therefore, estimate that the potential range of persistent yield impacts attributable to BC increasing SOC (and the associated effects on CEC and WHC) lies within the 50% of the yield-response data represented by the interquartile range (Supplementary Fig. 5).

### Current food prices

Supplementary Table 5 shows the production, yield and price of major food crops globally during the period 2008–2013, ranked by area harvested (derived from FAOStat data^59^). The range of impacts of BC on yields of major crops is shown in Supplementary Fig. 5. Note that major crops, such as sugar cane, that have shown either no significant yield improvements or only yield reductions with BC are not included, because it was assumed that BC would not be used for such crops. The economic values of these yield increments are then calculated by multiplying by the crop price. The interquartile range of these values for major crops with demonstrated yield responses to BC are shown in Supplementary Table 6 (based on inter-annual and international producer price fluctuations over the period 2008–2013).

Thus, depending on crop, soil and location the estimated value of increased crop yields (*R*_f,bc_) resulting from BC-SOC varies between −$13 to $63 per yr Mg^−1^ C (IQR), based on 2008–2013 crop prices. The NPV in perpetuity is then calculated as the sum of these annual returns discounted at the discount rate and diminishing exponentially in proportion to the decay rate of the BC (equation (13)).


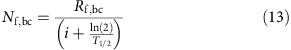


### Future food price trends

Projections of future food price trends have advanced considerably in recent years with an increasing diversity of trade models being applied under a range of possible projected trends in yield, and prices disaggregated by specific commodities, and under a range of future population and climate scenarios^60^,^61^,^62^,^63^,^64^,^65^. Existing studies point to a high degree of uncertainty in future food prices, and high sensitivity to assumptions about climate change, adaptation to climate change, CO_2_ fertilization, potential for yield improvements, dietary shifts, population, land degradation and competing land uses. Furthermore, there is a lack of published projections beyond 2050, giving rise to even greater uncertainty in trends through to 2100. Departing from the trend of falling food prices over the 20th century, current projections suggest that increasing population and affluence will drive an increase in food prices over the 21st century that will outstrip the potential for increased agricultural intensification to provide a counterbalance^64^,^65^. General and partial equilibrium trade models suggest that food price increases ranging from 3–84% are very likely by 2050 (refs 60, 61, 62, 63, 64, 65, 66). In the shorter term, we assume that food prices will increase by 3–5% in real terms by 2020 relative to 2000 (0–3% relative to current prices)^65^. In the absence of published estimates through to 2100, we make the simplifying assumption that world population increases from 2050 to 2100 by 14% (ref. 67), and would drive a proportional increase in food prices over this time interval. Thus we assume food prices rise by 4–96% relative to present values by 2100.

### Value of BC liming potential

In addition to BC’s potential to provide long lasting impacts on soil fertility through increased SOC, transient impacts of BC as a liming agent and organic fertilizer also have value. The economic value of BC’s impact on soil pH was estimated according to the method of ref. 14, whereby BC’s calcium carbonate equivalence (CCE_bc_) is given by equation (14). The NPV_cce_ of this liming potential is then calculated as the avoided cost of an equivalent quantity of agricultural lime, assuming a price of $10–80 Mg^−1^ CaCO_3_.


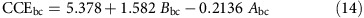


Where, *A*_bc_=ash content of the BC, and *B*_bc_=the percentage of base elements (Ca, Mg, K and Na) in the BC.

Phyllis 2 database^68^ gives mean ash content of straw, wood and bagasse as 5.5+/− 5.3% (1 s.d.), and base elements make up 41 +/− 34% (1 s.d.) of the ash elements by mass. To calculate ash content of the BC, it was assumed that ash was conserved during pyrolysis. The low end of the sensitivity range for liming value was set at zero, to represent situations in which the soil has no pH constraints, or when combustion ash from a BES might be used to provide an equivalent liming value. It was assumed that BC would not be used in situations that cause adverse pH impacts (for example, adding high pH BC to already alkaline soils).

### Value of BC nutrient content

A substantial fraction of biomass nitrogen can be volatilized during pyrolysis, predominantly as N_2_ (ref. 69). The nitrogen remaining in BC typically has negligible availability^70^. Therefore, no value was assumed for BC as a nitrogen fertilizer. Up to a maximum of 100% of biomass phosphorus and potassium was assumed to remain available in the BC. The low end of the sensitivity range for phosphorus and potassium fertilization value was set at zero, to represent situations in which combustion ash from a BES might be used to provide an equivalent nutrient value to BC. At the high end, lignocellulosic feedstocks of straw, wood or bagasse contain up to 800 mg P kg^−1^ and 9,000 mg K kg^−1^ (95% confidence)^68^. Nutrient use efficiency can be approximately 20% lower for organic than mineral fertilizers^71^,^72^, therefore, we base nutrient values on 80% of the equivalent prices of phosphorus in Ca(H_2_PO_4_)_2_ (triple super phosphate) and potassium in KCl. We use the 2005–2014 price ranges of $220–860 Mg^−1^ triple super phosphate and $180–650 Mg^−1^ KCl (ref. 73).

### BC haulage and field operations

It was estimated that BC haulage would cost between $0.26 to $14.17 per Mg feedstock (note that costs would be approximately four times greater per unit mass of BC), and that associated CO_2_–C emissions would be in the range 0.02–1.3 kg C per Mg feedstock, calculated as shown in Supplementary Table 11, and equations (15, 16, 17, 18, 19). Calculation of mean rectilinear distance (RD) between BEBCS plant and sites for application of BC followed equations (15, 16, 17) below:


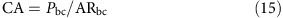



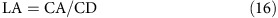



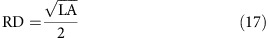


Where, CA is the area of cropland amended per year; *P*_bc_ is annual BC production; AR_bc_ is the application rate (mass per unit area) of BC on cropland; LA is the geographic area over which BC application is dispersed (including both BC-amended cropland and other intervening land where BC is not applied); and CD is the cropland density (area of cropland per unit area of landscape).

Then, haulage emissions (*H*_E_) and haulage cost (*H*_C_) per unit biomass feedstock are given by equations (18 and 19):


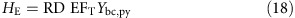



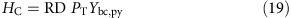


Where *Y*_bc,py_ is the yield of BC, and EF_T_ and *P*_T_ are the haulage emissions and costs, respectively, per unit distance per Mg of BC.

Field operations to incorporate BC may in some cases coincide with or substitute for normal tillage and spreading operations (for example, if BC is applied instead of agricultural lime). Therefore, we use zero cost as the low end of the sensitivity range for field operations. At the high end, field operations were estimated to cost up to $25 ha^−1^ for spreading, and $24 ha^−1^ for cultivation and incorporation (including labour, fuel, lubricant, and tractor and implement overheads) in cases when field operations cannot be combined with normal operations (for example, in no-till systems) and when it is not possible to use idle machinery at off-peak times^74^,^75^.

### Pyrolysis mass and energy balance

Pyrolysis mass and energy balances were calculated according to the method in ref. 10 (equations (1, 2, 3, 4, 5, 6, 7, 8, 9, 10, 11, 12, 13, 14) in Supporting Information of ref. 10). Pyrolysis temperature was in the range 450–650 °C. Process heat was assumed to be provided by combustion of a fraction of the combined gaseous and volatile pyrolysis products, with the remaining pyrolysis gases and volatiles used as fuel for power generation. Conversion efficiency of the power generation stage was assumed to be equal to that in the BES system. Parasitic power requirements were assumed to be provided by a fraction of the electricity generated.

### BC decomposition

Molar O:C ratio was used as a predictor variable of BC mineralization rate^76^. Data relating O:C ratio to estimated half-life were taken from the metastudy by ref. 76 (Supplementary Fig. 2). The regression of equation (20) thus yielded a value for the mineralization factor (*f*_d_) of 4.89 +/− 1.8 (1 s.d.). Mineralization rate in the Monte Carlo sensitivity analysis was, therefore, modelled using equation (20) with values of *f*_d_ populated from a Gaussian distribution with mean=4.89, and s.d.=1.8. O:C ratio was calculated according to equations (6, 7, 8) in the supporting information of ref. 12. It was assumed that carbon credits would be applicable to BC carbon remaining after 100 years.


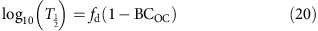


### N_2_O emissions from soil

Numerous studies have shown reduced N_2_O emissions from soil with BC application^77^. Reduction in soil N_2_O emissions is correlated with BC application rate, with the relative reduction varying between 0.17 to 0.91% of initial emissions per Mg ha^−1^ BC applied^77^. Unamended N_2_O emissions were calculated assuming the IPPC default N_2_O–N emission factor of 1.25% of applied fertilizer N, and N application rates to cropland were assumed to be in the range 25–200 kg N ha^−1^ per year^78^. (Supplementary Table 12).

Mechanisms and long-term dynamics of BC’s N_2_O impacts are still poorly understood and quantified. Some evidence suggests that N_2_O suppression could be short-lived, decaying over a few years^79^. We use a sensitivity range of 1–100 year for the time over which N_2_O suppression persists, with a logarithmic distribution to skew probability towards the lower end of this range.

### SOC feedback

BC can promote mineralization (an interaction sometimes referred to as ‘positive priming’) of easily mineralizable non-pyrogenic soil organic carbon (npSOC), and can also promote stabilization of npSOC, potentially leading to increased npSOC stocks in the long term (negative priming)^21^. Considerable uncertainty remains in the underlying mechanisms for both positive and negative priming, and in the sizes and longevity of these impacts under differing edaphic and environmental conditions^22^,^23^,^80^. Nonetheless, the ranges of published measurements of the magnitude of BC’s priming effect on npSOC turnover and stabilization can be used to modify rate constants in established SOC turnover models to estimate the possible range of priming impacts on long term npSOC stocks^22^. Applying this methodology to the RothC soil carbon model, while accounting also for impacts of BC on NPP, indicates that BC could potentially alter npSOC stocks over 100 years by up to −0.03 to +0.3 Mg npSOC per Mg BC C added. We adopt this method from Woolf and Lehmann^22^, applied over a 100 year time frame, to estimate the potential impact on npSOC within the sensitivity range of −0.03 to +0.3 mg npSOC per mg BC-C. The net magnitude of BEBCS sequestered carbon was then adjusted by adding the (positive or negative) npSOC feedback to the quantity of BC remaining in the soil. We note, however, that the value of any possible increase in npSOC may be challenging to internalize into carbon markets unless significant advances in understanding of the underlying mechanisms allow them to be predicted within tighter confidence limits.

### Code availability

Computer code to reproduce these results is available under GNU General Public License Version 3, at https://github.com/domwoolf/nets1.

### Data availability

All data required to repeat this experiment are provided within the article and the Supplementary Information. The data are also included in the computer code available for download (see Code Availability).

## Additional information

**How to cite this article:** Woolf, D. *et al*. Optimal bioenergy power generation for climate change mitigation with or without carbon sequestration. *Nat. Commun.* **7,** 13160 doi: 10.1038/ncomms13160 (2016).

## Supplementary Material

Supplementary Information Supplementary Figures 1-5, Supplementary Tables 1-12 and Supplementary References
