# Supplementary material for: Optimal bioenergy power generation for climate change mitigation with or without carbon sequestration
Source: Nat Commun. 2016 Oct 21;7:13160. doi: 10.1038/ncomms13160 (PMC5477486; doi:10.1038/ncomms13160)
Supplement: Supplementary Information — Supplementary Figures 1-5, Supplementary Tables 1-12 and Supplementary References [file ncomms13160-s1.pdf]

SUPPLEMENTARY FIGURES

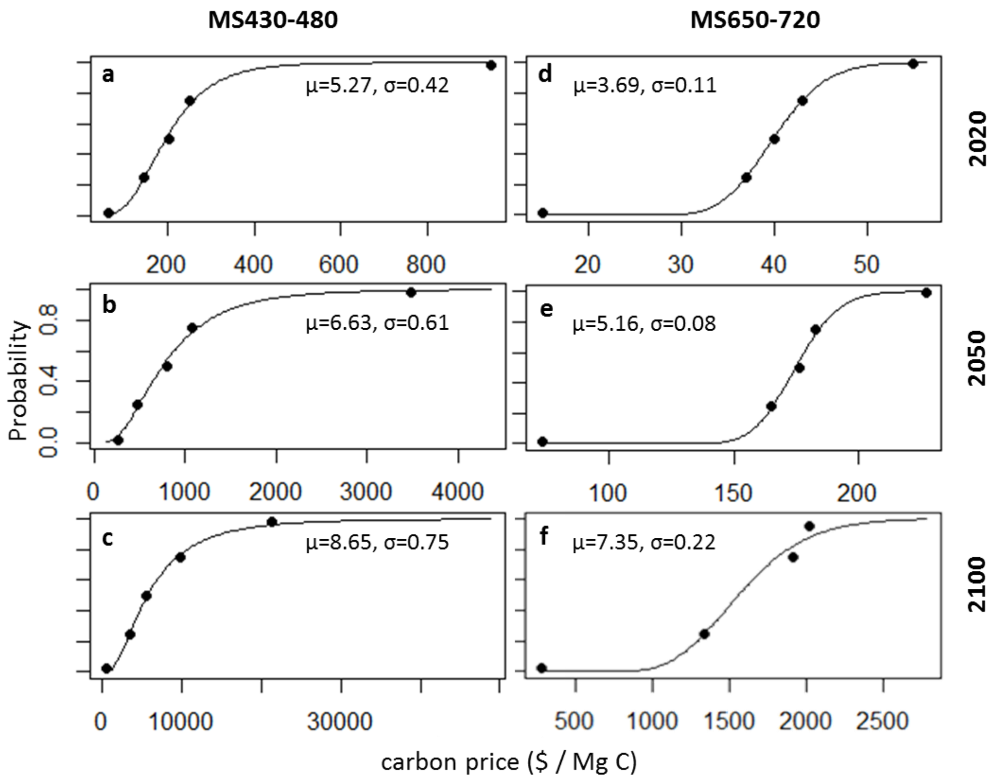

Supplementary Figure 1: Cumulative probability functions of carbon price for mitigation scenarios. Panels a-c show mitigation scenario MS430-480 and panels d-f are mitigation scenario MS650-720, in the years 2020 (a,c), 2050 (b,e), and 2100 (c,f). Probability functions are fitted to carbon price quartiles in the IPCC 5th Assessment Report[1] (Supplementary Table 1). μ and σ are the mean and standard deviation, respectively, of the natural-logarithm transformed carbon prices. The quantiles for a given year and concentration pathway were fitted to lognormal distributions using function get.lnorm.par in "rriskDistributions" of the R statistical programming language[2].

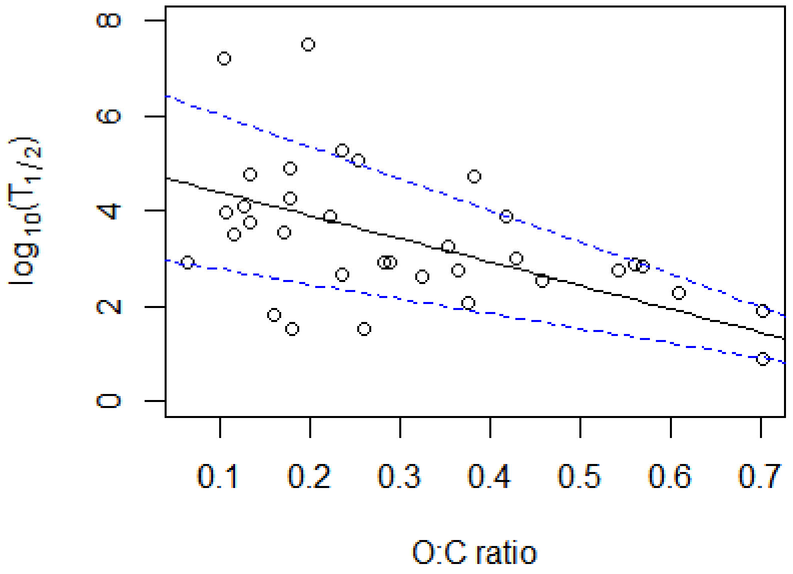

Supplementary Figure 2: Relationship between biochar molar O:C ratio and half life of the biochar in soil. Data from metastudy by Ref. 3. Dashed lines show one standard deviation.

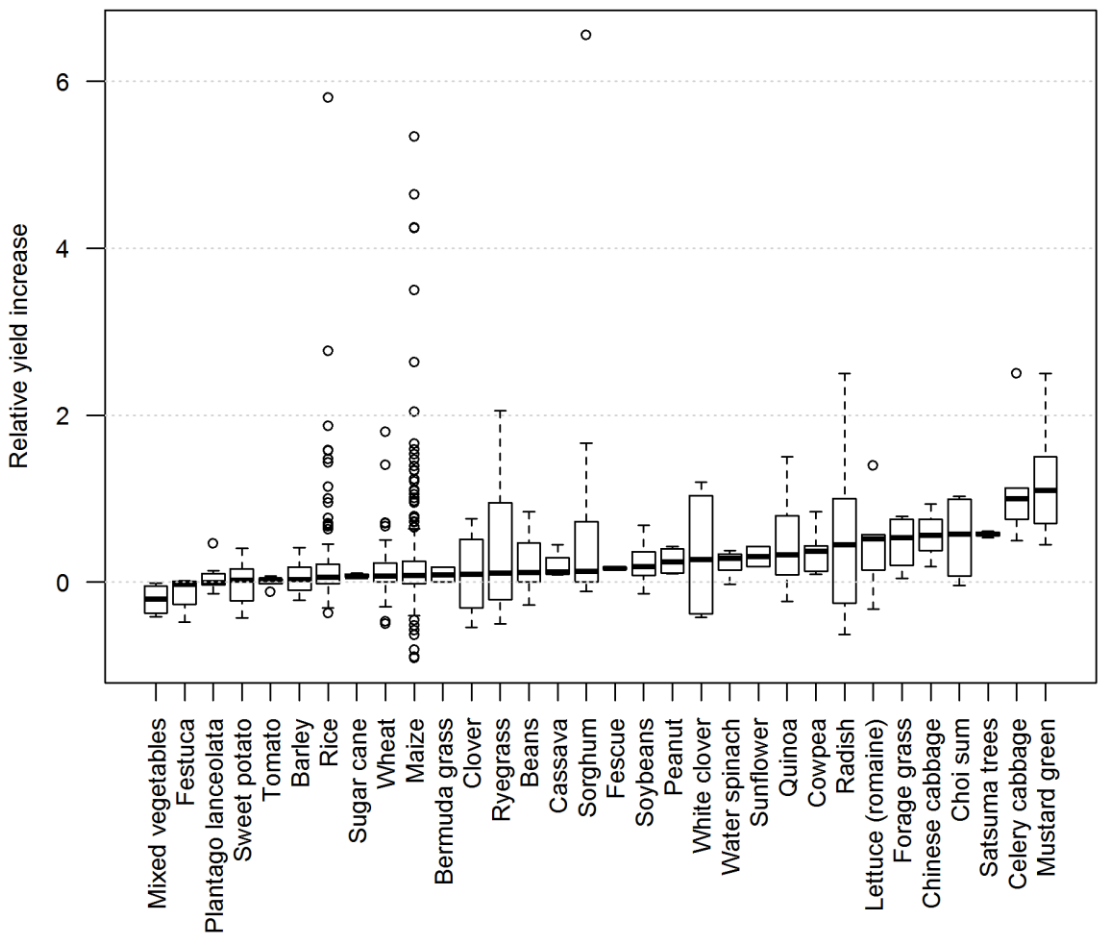

Supplementary Figure 3: Relative increase in crop yield from biochar addition. Relative increase is defined as the difference in yield between treatment and control, expressed as a fraction of control yield. Data from Refs. 4–75.

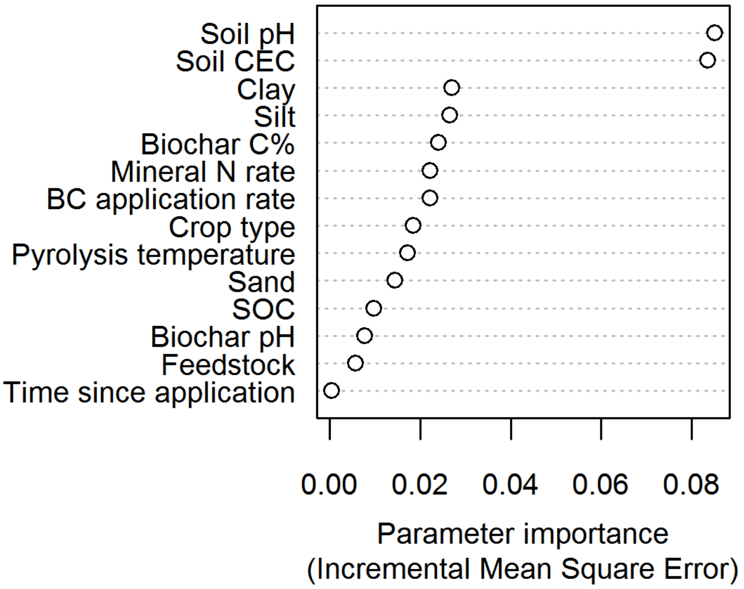

Supplementary Figure 4: Relative importance of model parameters in determining the relative crop yield increase from biochar additions. Relative importance was estimated by the incremental change in mean square error for each parameter in a random forest analysis.

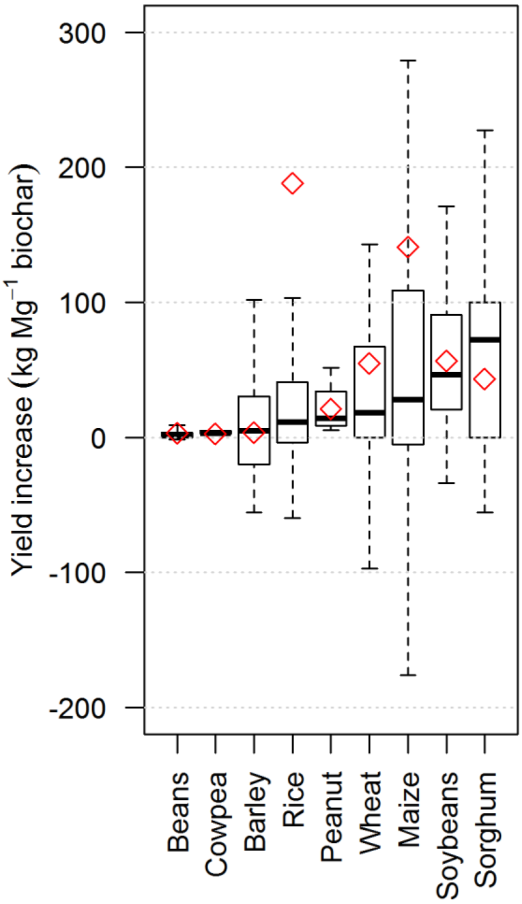

Supplementary Figure 5: Incremental change in annual crop yield per Mg of applied biochar, for major field crops for which biochar studies are published. Diamonds indicate means. Outliers not shown. Note that, the ranges of biochar-yield impacts are comparable to studies indicating that, for every 1 Mg ha^{-1} increase in SOC in the root zone of soils with low (< 2 %) initial SOC, crop yields can be increased by 20–70 kg ha^{-1} (wheat), 10–50 kg ha^{-1} (rice), and 30–300 kg ha^{-1} (maize).[76–86]

SUPPLEMENTARY TABLES

Supplementary Table 1: Carbon prices. Range of carbon prices in the IPCC WG III AR5 Scenario Database required to meet various atmospheric CO_2 eq. concentration targets in 2100.[87] The 430-480 ppm concentration pathway is compatible with representative concentration pathway RCP 2.6. Derived from Fig. 6.21a in Ref. 87. Note that values are given in 2014 USD per Mg carbon. To convert to prices per Mg CO_2, these should be divided by a factor of 3.67.

Year	Concentration pathway (ppm)	Carbon price ($ Mg^{-1} C)				
		Min	Lower quartile	Median	Upper quartile	Max
2020	650-720	15	37	40	40	55
	580-650	11	29	40	70	125
	530-580	15	51	88	143	990
	480-530	22	77	125	183	411
	430-480	62	143	202	249	946
2030	650-720	29	59	66	70	88
	580-650	15	51	77	125	312
	530-580	18	106	169	268	1,470
	480-530	59	154	220	370	737
	430-480	99	246	330	466	1,584
2050	650-720	73	165	176	183	227
	580-650	55	161	282	528	1,056
	530-580	48	271	524	748	3,032
	480-530	158	400	634	1,195	2,970
	430-480	264	473	799	1,085	3,483
2100	650-720	279	1,335	1,870	1,943	1,991
	580-650	359	799	1,745	2,105	5,724
	530-580	125	788	1,777	5,306	10,083
	480-530	473	3,458	5,473	9,090	30,855
	430-480	455	3,458	5,586	9,739	21,252

Supplementary Table 2: Parameter abbreviations, units and distribution types.

Category	Parameter	Abbreviation	Units	Distribution type
**Common Parameters for all conversion technologies**				
Economic	discount rate	Disc. rate	dimensionless	Uniform
	biomass ash content	BM ash	dimensionless	Uniform
	crop price	Crop price	$ Mg^{-1}	Uniform
	agricultural lime price	Lime price	$ Mg^{-1}	Uniform
	carbon price	C price	$ Mg^{-1}	Log-normal
	electricity price	Elec. price	$ GJ^{-1}	Uniform
**Reference Energy System**				
	C intensity	C intensity	Mg C GJ^{-1} f	Uniform
	electrical conversion efficiency	FF eff.	GJ GJ^{-1} f	Uniform
**BES Parameters**				
Economic	capital cost	BES CC	$ (Mg f yr^{-1})$^{-1}$	Uniform
	life time of plant	BES life	yr	Uniform
Energy	electrical conversion efficiency	BES eff.	GJ GJ^{-1} f	Uniform
**BEBCS Parameters**				
Pyrolysis	pyrolysis temperature	Py. temp.	°C	Uniform
Energy	electrical conversion efficiency	BEBCS eff.	GJ GJ^{-1} f	Uniform
Economic	capital cost	BEBCS CC	$ (Mg f yr^{-1})$^{-1}$	Uniform
	life time of plant ratio to BES	BEBCS life ratio	Dimensionless	Uniform
	biochar haulage cost	BC haul. cost	$ Mg^{-1} f	Uniform
	biochar field operations	BC field cost	$ ha^{-1}	Uniform
GHG feedbacks	unamended N additions	N app. rate	kg ha^{-1} yr^{-1}	Uniform
	N_2O feedback factor	N2O factor	%N_2O (Mg BC ha^{-1})$^{-1}$	Uniform
	years of N_2O effect	N2O years	yr	Log
	SOC feedback factor	SOC factor	dimensionless	Uniform
	biochar haulage CO_2	BC haul. CO2	Mg C Mg^{-1} f	Uniform
Soil	biochar nutrient value	BC nutrient	$ Mg^{-1} f	Uniform
	biochar yield impact	BC yield impact	$ yr^{-1} Mg^{-1} BC	Uniform
	biochar stability factor	BC stab. fact.	dimensionless	Normal
**BECCS Parameters**				
Economic	cost of CCS	CCS cost	$ Mg^{-1} C captured	Uniform
	efficiency penalty (rel BES)	CCS eff. penalty	dimensionless	Uniform
	CO_2 sequestration fraction	CCS seq. fraction	dimensionless	Uniform

Supplementary Table 3: Parameter ranges for mitigation scenario MS430-480.

	MS430-480, 2020				MS430-480, 2050				MS430-480, 2100			
**Parameter**	**Min**	**Max**	**Mean**	**S.D**	**Min**	**Max**	**Mean**	**S.D**	**Min**	**Max**	**Mean**	**S.D**
Disc. rate	0.015	0.06	0.03	0.013	0.015	0.06	0.03	0.013	0.015	0.06	0.03	0.013
BM ash	0.002	0.108	0.055	0.031	0.002	0.108	0.055	0.0306	0.002	0.108	0.055	0.031
Crop price	247.2	423.2	335.2	50.8	247.2	741.5	494.4	142.7	281.8	845.3	335.2	162.7
Lime price	10	80	45	20	10	80	45	20	10	80	45	20
C price[1]			5.27	0.42			6.63	0.61			8.65	0.75
Elec. price	16.4	63.1	39.7	13.5	26.2	100.9	63.5	21.6	52.3	201.8	39.7	43.1
C intensity	0.0E+00	1.9E-02	9.5E-03	5.5E-03	0.0E+00	1.9E-02	9.5E-03	5.5E-03	0.0E+00	1.9E-02	9.5E-03	5.5E-03
FF eff.	0.38	0.5	0.44	0.035	0.38	0.5	0.44	0.035	0.38	0.5	0.44	0.035
BES CC	402	1029	716	181	316	846	581	153	316	846	716	153
BES life	25	50	38	7	25	50	38	7	25	50	38	7
BES eff.	0.3	0.42	0.36	0.035	0.33	0.45	0.39	0.035	0.33	0.45	0.36	0.035
Py. temp.	450	650	550	58	450	650	550	58	450	650	550	58
BEBCS eff.	0.30	0.42	0.45	0.03	0.33	0.45	0.45	0.03	0.33	0.45	0.45	0.03
BEBCS CC	327	763	545	126	235	512	374	80	235	512	545	80
BEBCS life ratio	1	1	1	0	1	1	1	0	1	1	1	0
BC haul. cost	0.3	15.9	8.1	4.5	0.5	25.4	12.9	7.2	0.9	50.8	8.1	14.4
BC field cost	0.00	54.88	27.44	15.84	0.00	87.81	43.90	25.35	0.00	175.62	27.44	50.70
N app. rate	25.00	200.00	100.00	50.52	25.00	200.00	100.00	50.52	25.00	200.00	100.00	50.52
N2O factor	1.7E-3	1.5E-2	8.6E-3	3.9E-3	1.7E-3	1.5E-2	8.5E-3	3.9E03	1.7E-3	1.5E-2	8.5E03	3.9E-3
N2O years	1.0	100.0	21.0	24.6	1.0	100.0	21.0	24.6	1.0	100.0	21.0	24.6
SOC factor	-0.03	0.3	0.135	0.095	-0.03	0.3	0.135	0.095	-0.03	0.3	0.135	0.095
BC haul. CO2	2.2-5	1.3E-3	6.7E-4	3.7E-4	2.2E-5	1.32E-3	6.7E-4	3.74E-4	2.2E-5	1.32E-3	6.70E-4	3.74E-4
BC nutrient	0.00	16.49	8.25	4.76	0.00	16.49	8.25	4.76	0.00	16.49	8.25	4.76
BC yield impact	-13.33	65.93	26.30	22.88	-13.33	115.53	51.10	37.20	-13.46	123.07	26.30	39.41
BC stab. fact.			4.89	1.8			4.89	1.8			4.89	1.8
CCS cost	183	470	327	83	183	470	327		183	470	327	83
CCS eff. penalty	0.04	0.11	0.075	0.020	0.04	0.11	0.075		0.04	0.11	0.075	0.020
CCS seq. fraction	0.81	0.91	0.86	0.029	0.81	0.91	0.86		0.81	0.91	0.86	0.029

[a] For log-normal distributions, mean and standard deviations are of the natural-logarithm transformed values.

Supplementary Table 4: Parameter ranges for mitigation scenario MS650-720.

	MS650-720, 2020				MS650-720, 2050				MS650-720, 2100			
**Parameter**	**Min**	**Max**	**Mean**	**S.D**	**Min**	**Max**	**Mean**	**S.D**	**Min**	**Max**	**Mean**	**S.D**
Disc. rate	0.015	0.060	0.030	0.013	0.015	0.06	0.03	0.013	0.015	0.06	0.03	0.013
BM ash	0.002	0.108	0.055	0.03	0.002	0.108	0.055	0.03	0.002	0.108	0.055	0.03
Crop price	247.2	423.2	335.2	50.8	247.2	741.5	335.2	142.7	281.8	845.3	335.2	162.7
Lime price	10	80	45	20	10	80	45	20	10	80	45	20
C price[1]			3.69	0.61			5.16	0.08			7.35	0.22
Elec. price	16.35	63.06	39.70		26.16	100.89	39.70	21.57	52.33	201.78	39.70	43.14
C intensity	0.0E+0	1.9E-2	9.5E-3	5.5E-3	0.0E+0	1.9E-2	9.5E-3	5.5E-3	0.0E+0	1.9E-2	9.5E-3	5.5E-3
FF eff.	0.380	0.500	0.440	0.035	0.38	0.5	0.44	0.035	0.38	0.5	0.44	0.035
BES CC	402	1029	716	181	316	846	716	153	316	846	716	153
BES life	25	50	38	7	25	50	38	7	25	50	38	7
BES eff.	0.300	0.420	0.360	0.035	0.33	0.45	0.36	0.035	0.33	0.45	0.36	0.035
Py. temp.	450	650	550	58	450	650	550	58	450	650	550	58
BEBCS eff.	0.30	0.42	0.45	0.03	0.33	0.45	0.45	0.03	0.33	0.45	0.45	0.03
BEBCS CC	279	650	465	107	235	512	545	80	235	512	545	80
BEBCS life ratio	1	1	1	0	1	1	1	0	1	1	1	0
BC haul. cost	0.3	15.9	8.1	4.5	0.5	25.4	8.1	7.2	0.9	50.8	8.1	14.4
BC field cost	0.00	54.88	27.44	15.84	0.00	87.81	27.44	25.35	0.00	175.62	27.44	50.70
N app. rate	25.00	200.00	100.00	50.52	25.00	200.00	100.00	50.52	25.00	200.00	100.00	50.52
N2O factor	1.7E-3	1.5E-2	8.5E-3	3.9E-3	1.7E-3	1.5E-2	8.5E-3	3.9E-3	1.7E-3	1.5E-2	8.5E-3	3.9E-3
N2O years	1.0	100.0	50.5		1.0	100.0	21.0	24.6	1.0	100.0	21.0	24.6
SOC factor	-0.030	0.300	0.135	0.095	-0.03	0.3	0.135	0.095	-0.03	0.3	0.135	0.095
BC haul. CO2	2.2E-5	1.3E-3	6.7E-4	3.7E-4	2.2E-5	1.3E-3	6.7E-4	3.7E-4	2.2E-5	1.3E-3	6.7E-4	3.7E-4
BC nutrient	0.00	16.49	8.25	4.76	0.00	16.49	8.25	4.76	0.00	16.49	8.25	4.76
BC yield impact	-13.33	65.93	26.30	22.88	-13.33	115.53	26.30	37.20	-13.46	123.07	26.30	39.41
BC stab. fact.			4.89	1.8			4.89	1.8			4.89	1.8
CCS cost	183	470	327	83	183	470	327 83		183	470	327	83
CCS eff. penalty	0.040	0.110	0.075	0.020	0.04	0.11	0.075 0.020		0.04	0.11	0.075	0.020
CCS seq. fraction	0.810	0.910	0.860	0.029	0.81	0.91	0.86 0.029		0.81	0.91	0.86	0.029

[a] For log-normal distributions, mean and standard deviations are of the natural-logarithm transformed values.

Supplementary Table 5: Production, yield and price of major food crops globally, ranked by area harvested (2008-2013 data). Yields are the global mean values. Prices are weighted means of producer prices in each country, weighted by production quantity. Data derived from FAOStat.[88]

Crop	Area harvested [a] (Mha)	Production [a] (Tg)	Yield [b] (Mg ha^{-1})	Price [c] ($ Mg^{-1})	Value per ha [c] ($ ha^{-1})
Wheat	218	713	3.3	254	828
Maize	184	1,017	5.5	239	1,318
Rice, paddy	165	746	4.5	403	1,824
Soybeans	111	276	2.5	623	1,548
Barley	50	145	2.9	223	647
Sorghum	42	61	1.5	310	452
Seed cotton	37	73	2.0	781	1,545
Rapeseed	36	73	2.0	589	1,175
Millet	33	30	0.9	383	347
Beans, dry	29	23	0.8	1,685	1,333
Sugar cane	27	1877	70.8	296	20,949
Sunflower	26	45	1.7	633	1,108
Groundnuts	25	45	1.8	1,384	2,460
Cassava	21	277	13.3	451	6,025
Potatoes	19	368	18.9	418	7,909
Oil palm	17	268	15.7	152	2,391
Chick peas	14	13	1.0	1,084	1,049
Coconuts	12	62	5.1	339	1,730
Cow peas, dry	11	6	0.5	1,762	890
Olives	10	20	2.0	1,101	2,186
Oats	10	24	2.4	194	473
Sesame seed	9	5	0.5	1,550	784
Sweet potatoes	8	111	13.4	554	7,449
Peas, dry	6	11	1.7	684	1,177
Pigeon peas	6	5	0.8	1,277	974
Pulses, nes	6	5	0.8	1,463	1,229
Rye	6	17	2.9	204	590
Yams	5	60	11.9	1,057	12,586
Sugar beet	4	250	56.3	99	5,595
Lentils	4	5	1.1	1,087	1,238
Triticale	4	15	3.8	192	728
Other Cereal	8	28	2.4	202	479

[a] World total

[b] World mean

[c] Weighted mean (weighted by production quantity per country)

Supplementary Table 6: Interquartile ranges of the economic value of biochar yield impacts for major crops with demonstarted positive reponses to biochar. Based on current (2008-2013) inter-annual and international crop price fluctuations.[88]

	Yield increment (kg crop yr^{-1} Mg^{-1} C)		Crop Price (\$ Mg^{-1})		Value of yield increment (\$ yr^{-1} Mg^{-1} C)	
	Lower quartile	Upper quartile	Lower quartile	Upper quartile	min	max
Wheat	0	67	231	368	0.00	24.66
Maize	-5	109	224	335	-1.68	36.52
Rice	-4	41	309	623	-2.49	25.54
Sorghum	0	100	215	392	0.00	39.20
Barley	-20	31	223	647	-12.94	20.06
Peanut	9	34	714	1382	6.43	46.99
Beans	0	4	802	1,860	0.00	7.44
Cowpea	3	5	677	1,567	2.03	7.84
Soybean	21	91	510	690	10.71	62.79
Range					-12.94	62.79

Supplementary Table 7: Fraction of primary energy from low-carbon sources in RCP 2.6 [a]

	2010	2030	2050	2100
lower quartile	0.138	0.23	0.54	0.9
median	0.15	0.25	0.6	0.92
upper quartile	0.186	0.31	0.66	0.95

[a] IPCC 5th Assessment Report (2014), Working Group 3, Ch. 7, Fig 7.14, p560 [89]

Supplementary Table 8: Estimated carbon intensity (Mg C GJe^{-1}) of power generation in RCP 2.6.

	2010	2030	2050	2100
mean	0.040	0.035	0.019	0.004
IQR	0.004	0.004	0.006	0.002
s.d.	0.003	0.003	0.004	0.002

Supplementary Table 9: Power generation efficiency and capital costs for bioenergy in 2012 and projections for 2030. Data ranges are from Refs.90–95.

Year		Capacity (MW)				
		<10	10 - 50	>50	Co-firing[a]	Co-firing[b]
2012	Power generation efficiency (%)	14-18	18-33	28-40	35-39	35-39
	Capital costs (USD/kW)	6000-9800	3900-5800	2400-4200	300-700	3200-4000
	Operating costs (% of capital costs)	5.5-6.5	5-6	3-5	2.5-3.5	2.5-3.5
2030	Power generation efficiency (%)	16-20	23-38	33-45	33-45	33-45
	Capital costs (USD/kW)	4800-7800	3100-4600	1900-3400	300-700	3200-4000
	Operating costs (% of capital costs)	5.5-6.5	5-6	3-5	2.5-3.5	2.5-3.5

[a] Co-firing costs related only to the investment in additional systems for handling the biomass fuels, with no contribution to the costs of the coal-fired plant itself.

[b] Total co-firing cost inclusive of cost of the coal fired plant.

Supplementary Table 10: Carbon capture and storage costs.

	Minimum cost ($ Mg^{-1} CO_2 captured)	Maximum cost ($ Mg^{-1} CO_2 captured)
Published fossil fuel CCS cost	49	80
Capacity factor adjustment	0	14
Supplemental CO_2 transport cost for BECCS	0	25
Net biomass CCS cost	49	119

Supplementary Table 11: Biochar haulage costs and emissions

	Symbol	Units	low end estimate	high end estimate
Plant size	P	MW	10	250
Biochar production	P_{bc}	Mg yr^{-1}	3429	85715
Mean biochar application rate	AR_{bc}	Mg ha^{-1} yr^{-1}	2.0	0.2
Cropland area	CA	ha	1714	428577
Cropland density	CD	ha/ha	0.90	0.25
Total land area	LA	km^2	19	17143
Mean rectilinear distance	RD	km	2.2	65.5
Haulage cost	H_{Cd}	\$ Mg^{-1} km^{-1}	0.50	0.90
Haulage cost	H_C	\$ Mg^{-1} f	0.26	14.17
Haulage emissions	H_{Ed}	Mg C Mg^{-1} km^{-1}	4.18E-05	8.36E-05
Haulage emissions	H_E	Mg C Mg^{-1} f	2.2E-05	1.32E-03

Supplementary Table 12: Soil N_2O emissions parameter ranges.

	Min	Max
N_2O suppression factor (% Mg^{-1} ha^{-1})	0.17	0.85
Initial fertilizer-N application rate (kg N ha^{-1} yr^{-1})	25	200
Length of N_2O suppression (yr) (log-normal distribution)	1	100

SUPPLEMENTARY REFERENCES

1. Clarke, L. *et al.* in *Climate Change 2014: Mitigation of Climate Change. Contribution of Working Group III to the Fifth Assessment Report of the Intergovernmental Panel on Climate Change* (eds Edenhofer, O., R. *et al.*) 413-510 (Cambridge University Press, 2014).
2. R Development Core Team. *R: A language and environment for statistical computing*. Available at http://www.r-project.org (2008).
3. Kurt A Spokas. Review of the stability of biochar in soils: predictability of O:C molar ratios. *Carbon Manag.* **1,** 289–303 (2010).
4. Aguilar-Chávez, Á., Díaz-Rojas, M., Cárdenas-Aquino, M. del R., Dendooven, L. & Luna-Guido, M. Greenhouse gas emissions from a wastewater sludge-amended soil cultivated with wheat (Triticum spp. L.) as affected by different application rates of charcoal. *Soil Biol. Biochem.* **52,** 90–95 (2012).
5. Arif, M. *et al.* Effect of biochar FYM and mineral nitrogen alone and in combination on yield and yield components of maize. *Sarhad J Agric* **28,** 191–195 (2012).
6. Artiola, J. F., Rasmussen, C. & Freitas, R. Effects of a Biochar-Amended Alkaline Soil on the Growth of Romaine Lettuce and Bermudagrass: *Soil Sci.* **177,** 561–570 (2012).
7. Asai, H. *et al.* Biochar amendment techniques for upland rice production in Northern Laos: 1. Soil physical properties, leaf SPAD and grain yield. *Field Crops Res.* **111,** 81–84 (2009).
8. Baronti, S. *et al.* The Italian Biochar Initiative (ITABI): Effects on soil fertility and on crops production. Available at http://www.biochar-international.org/images/Baronti_poster_newcastle_C2.pdf (2008).
9. Blackwell, P. *et al.* Improving wheat production with deep banded oil mallee charcoal in Western Australia. in *First Asia Pacific Biochar Conference, Terrigal, Australia* **30,** (2007).
10. Blackwell, P., Krull, E., Butler, G., Herbert, A. & Solaiman, Z. Effect of banded biochar on dryland wheat production and fertiliser use in south-western Australia: an agronomic and economic perspective. *Soil Res.* **48,** 531–545 (2010).
11. Chan, K. Y., Van Zwieten, L., Meszaros, I., Downie, A. & Joseph, S. Agronomic values of greenwaste biochar as a soil amendment. *Aust. J. Soil Res.* **45,** 629–634 (2007).
12. Chan, K. Y., Van Zwieten, L., Meszaros, I., Downie, A. & Joseph, S. Using poultry litter biochars as soil amendments. *Aust. J. Soil Res.* **46,** 437–444 (2008).
13. Chen, Y., Shinogi, Y. & Taira, M. Influence of biochar use on sugarcane growth, soil parameters, and groundwater quality. *Soil Res.* **48,** 526–530 (2010).
14. Cornelissen, G. *et al.* Biochar effect on maize yield and soil characteristics in five conservation farming sites in Zambia. *Agronomy* **3,** 256–274 (2013).
15. Cui, L. *et al.* Biochar amendment greatly reduces rice Cd uptake in a contaminated paddy soil: A two-year field experiment. *BioResources* **6,** 2605–2618 (2011).
16. Cui, L. *et al.* The reduction of wheat Cd uptake in contaminated soil via biochar amendment: A two-year field experiment. *BioResources* **7,** 5666–5676 (2012).
17. Deal, C., Brewer, C. E., Brown, R. C., Okure, M. A. & Amoding, A. Comparison of kiln-derived and gasifier-derived biochars as soil amendments in the humid tropics. *Biomass Bioenergy* **37,** 161–168 (2012).

18. Dou, L., Komatsuzaki, M., Nakagawa, M. & others. Effects of Biochar, Mokusakueki and Bokashi application on soil nutrients, yields and qualities of sweet potato. *Int. Res. J. Agric. Sci. Soil Sci.* **2,** 318–327 (2012).
19. Gaskin, J. W. *et al.* Effect of Peanut Hull and Pine Chip Biochar on Soil Nutrients, Corn Nutrient Status, and Yield. *Agron. J.* **102,** 623 (2010).
20. Gathorne-Hardy, A. The role of biochar in English agriculture: agronomy, biodiversity, economics and climate change. *Thesis for the degree of Doctor of Philosophy* (Imperial College London, 2012).
21. Güereña, D., et al. Nitrogen dynamics following field application of biochar in a temperate North American maize-based production system. Plant Soil 365, 239-254 (2013).
22. Haefele, S. M. *et al.* Effects and fate of biochar from rice residues in rice-based systems. *Field Crops Res.* **121,** 430–440 (2011).
23. Hossain, M. K., Strezov, V., Chan, K. Y. & Nelson, P. F. Agronomic properties of wastewater sludge biochar and bioavailability of metals in production of cherry tomato (Lycopersicon esculentum). *Chemosphere* **78,** 1167–1171 (2010).
24. Hua, L., Chen, Y. & Wu, W. Impacts upon soil quality and plant growth of bamboo charcoal addition to composted sludge. *Environ. Technol.* **33,** 61–68 (2012).
25. Ishii, T. & Kadoya, K. Effects of charcoal as a soil conditioner on citrus growth and vesicular-arbuscular mycorrhizal development. *J. Jpn. Soc. Hortic. Sci. Jpn.* (1994).
26. Islam, M. N. & Ani, F. N. Techno-economics of rice husk pyrolysis, conversion with catalytic treatment to produce liquid fuel. *Bioresour. Technol.* **73,** 67–75 (2000).
27. Islami, T., Guritno, B., Basuki, N., Suryanto, A. & others. Biochar for sustaining productivity of cassava based cropping systems in the degraded lands of East Java, Indonesia. *J. Trop. Agric.* **49,** 40–46 (2011).
28. Jia, J., Li, B., Chen, Z., Xie, Z. & Xiong, Z. Effects of biochar application on vegetable production and emissions of N2O and CH4. *Soil Sci. Plant Nutr.* **58,** 503–509 (2012).
29. Jones, D. L. *et al.* Short-term biochar-induced increase in soil CO2 release is both biotically and abiotically mediated. *Soil Biol. Biochem.* **43,** 1723–1731 (2011).
30. Kammann, C. I., Linsel, S., Gö\s sling, J. W. & Koyro, H.-W. Influence of biochar on drought tolerance of Chenopodium quinoa Willd and on soil–plant relations. *Plant Soil* **345,** 195–210 (2011).
31. Kimetu, J. *et al.* Reversibility of Soil Productivity Decline with Organic Matter of Differing Quality Along a Degradation Gradient. *Ecosystems* **11,** 726–739 (2008).
32. Lehmann, J. *et al.* Nutrient availability and leaching in an archaeological Anthrosol and a Ferralsol of the Central Amazon basin: fertilizer, manure and charcoal amendments. *Plant Soil* **249,** 343–357 (2003).
33. Lentz, R. D. & Ippolito, J. A. Biochar and Manure Affect Calcareous Soil and Corn Silage Nutrient Concentrations and Uptake. *J. Environ. Qual.* **41,** 1033 (2012).
34. Liu, X. *et al.* Can biochar amendment be an ecological engineering technology to depress N2O emission in rice paddies?—A cross site field experiment from South China. *Ecol. Eng.* **42,** 168–173 (2012).

35. Luyen, P. T., Khang, D. N. & Preston, T. R. Effects of biochar from gasifier stove and effluent from biodigester on growth of maize in acid and fertile soils. **24,** (2012).
36. Major, J., Rondon, M., Molina, D., Riha, S. J. & Lehmann, J. Maize yield and nutrition during 4 years after biochar application to a Colombian savanna oxisol. *Plant Soil* **333,** 117–128 (2010).
37. Masulili, A., Utomo, W. H. & Syechfani, M. S. Rice husk biochar for rice based cropping system in acid soil 1. The characteristics of rice husk biochar and its influence on the properties of acid sulfate soils and rice growth in West Kalimantan, Indonesia. *J. Agric. Sci.* **2,** 39 (2010).
38. Mia, S. *et al.* Biochar application rate affects biological nitrogen fixation in red clover conditional on potassium availability. *Agric. Ecosyst. Environ.* **191,** 83–91 (2014).
39. Nehls, T. Fertility improvement of a Terra Firme Oxisol in central Amazonia by charcoal applications. *Thesis for the degree of PhD in Geoecology* (Univ. Bayreuth Inst. Soil Sci. Soil Geogr.) **81,** (2002).
40. Nguyen, H., Blair, G. & Guppy, C. Effect of rice husk biochar and nitrification inhibitor treated urea on N and other macronutrient uptake by maize. *Proceedings of 16th Australian Agronomy Conference* (2012).
41. Noguera, D. *et al.* Contrasted effect of biochar and earthworms on rice growth and resource allocation in different soils. *Soil Biol. Biochem.* **42,** 1017–1027 (2010).
42. Nzanza, B., Marais, D., Soundy, P. & others. Effect of arbuscular mycorrhizal fungal inoculation and biochar amendment on growth and yield of tomato. *Int. J. Agric. Biol.* **14,** 965–969 (2012).
43. Oguntunde, Fosu, Ajayi & Giesen. Effects of charcoal production on maize yield, chemical properties and texture of soil. *Biol. Fertil. Soils* **39,** 295–299 (2004).
44. Park, J. H., Choppala, G. K., Bolan, N. S., Chung, J. W. & Chuasavathi, T. Biochar reduces the bioavailability and phytotoxicity of heavy metals. *Plant Soil* **348,** 439–451 (2011).
45. Peng, X., Ye, L. L., Wang, C. H., Zhou, H. & Sun, B. Temperature- and duration-dependent rice straw-derived biochar: Characteristics and its effects on soil properties of an Ultisol in southern China. *Soil Tillage Res.* **112,** 159–166 (2011).
46. Pichler, B. Biochars and their impact on nutrient retention and water mass balance in a Swiss vineyard soil. (University of Zurich, 2010).
47. Quilliam, R. S. *et al.* Nutrient dynamics, microbial growth and weed emergence in biochar amended soil are influenced by time since application and reapplication rate. *Agric. Ecosyst. Environ.* **158,** 192–199 (2012).
48. Rajkovich, S. *et al.* Corn growth and nitrogen nutrition after additions of biochars with varying properties to a temperate soil. *Biol. Fertil. Soils* **48,** 271–284 (2012).
49. Rodríguez, L., Salazar, P. & Preston, T. R. Effect of a culture of 'native' micro-organisms, biochar and biodigester effluent on the growth of maize in acid soils. *Livest. Res. Rural Dev.* **23,** 1–7 (2011).
50. Rondon, M., Lehmann, J., Ramírez, J. & Hurtado, M. Biological nitrogen fixation by common beans (Phaseolus vulgaris L.) increases with bio-char additions. *Biol. Fertil. Soils* **43,** 699–708 (2007).

51. Schnell, R. W., Vietor, D. M., Provin, T. L., Munster, C. L. & Capareda, S. Capacity of biochar application to maintain energy crop productivity: Soil chemistry, sorghum growth, and runoff water quality effects. *J. Environ. Qual.* **41,** 1044–1051 (2012).
52. Shackley, S. *et al.* Sustainable gasification–biochar systems? A case-study of rice-husk gasification in Cambodia, Part II: Field trial results, carbon abatement, economic assessment and conclusions. *Energy Policy* **41,** 618–623 (2012).
53. Sokchea, H., Borin, K. & Preston, T. R. Effect of biochar from rice husks (combusted in a downdraft gasifier or a paddy rice dryer) on production of rice fertilized with biodigester effluent or urea. *Lives.t Res. Rural Dev.* **25,** (2013).
54. Solaiman, Z. M., Blackwell, P., Abbott, L. K. & Storer, P. Direct and residual effect of biochar application on mycorrhizal root colonisation, growth and nutrition of wheat. *Soil Res.* **48,** 546–554 (2010).
55. Steiner *et al.* Long term effects of manure, charcoal and mineral fertilization on crop production and fertility on a highly weathered Central Amazonian upland soil. *Plant Soil* **291,** 275–290 (2007).
56. Suddick, E. C. & Six, J. An estimation of annual nitrous oxide emissions and soil quality following the amendment of high temperature walnut shell biochar and compost to a small scale vegetable crop rotation. *Sci. Total Environ.* **465,** 298–307 (2013).
57. Sukartono, U. W., Kusuma, Z. & Nugroho, W. H. Soil fertility status, nutrient uptake, and maize (Zea mays L.) yield following biochar and cattle manure application on sandy soils of Lombok, Indonesia. *J. Trop. Agric.* **49,** 47–52 (2011).
58. Taghizadeh-Toosi, A. Ammonia and nitrous oxide emissions from soils under ruminant urine patches and the effects of biochar amendment on these emissions and plant nitrogen uptake. (Lincoln University, 2011).
59. Taghizadeh-Toosi, A. *et al.* Biochar incorporation into pasture soil suppresses in situ nitrous oxide emissions from ruminant urine patches. *J. Environ. Qual.* **40,** 468–476 (2011).
60. Tagoe, S., Horiuchi, T. & Matsui, T. Effects of carbonized and dried chicken manures on the growth, yield, and N content of soybean. *Plant Soil* **306,** 211–220 (2008).
61. Tatarková, V., Hiller, E. & Vaculík, M. Impact of wheat straw biochar addition to soil on the sorption, leaching, dissipation of the herbicide (4-chloro-2-methylphenoxy acetic acid) and the growth of sunflower (Helianthus annuus L.). *Ecotoxicol. Environ. Saf.* **92,** 215–221 (2013).
62. Chhay, T., Vor, S., Borin, K. & Preston, T. R. Effect of different levels of biochar on the yield and nutritive value of Celery cabbage (Brassica chinensis var), Chinese cabbage (Brassica pekinensis), Mustard green (Brassica juncea) and Water spinach (Ipomoea aquatica). *Livest. Res. Rural Dev.* **25,** (2012).
63. Uddin, S. M. M., Murayama, S., Ishimine, Y. & Tsuzuki, E. Studies on sugarcane cultivation. 1. Effect of the mixture of charcoal with pyroligneous acid on cane and sugar yield of spring and ratoon crops of sugarcane (Saccharum officinarum L.). *Jpn. J. Trop. Agr.* **38,** 281–285 (1994).
64. Vaccari, F. P. *et al.* Biochar as a strategy to sequester carbon and increase yield in durum wheat. *Eur. J. Agron.* **34,** 231–238 (2011).

65. Van Zwieten, L. *et al.* Effects of biochar from slow pyrolysis of papermill waste on agronomic performance and soil fertility. *Plant Soil* **327,** 235–246 (2010).
66. Wang, J., Pan, X., Liu, Y., Zhang, X. & Xiong, Z. Effects of biochar amendment in two soils on greenhouse gas emissions and crop production. *Plant Soil* **360,** 287–298 (2012).
67. Warnock, D. D. *et al.* Influences of non-herbaceous biochar on arbuscular mycorrhizal fungal abundances in roots and soils: Results from growth-chamber and field experiments. *Appl. Soil Ecol.* **46,** 450–456 (2010).
68. Wisnubroto, E. I. Investigation on the effect of biochar addition and the use of pasture species with different rooting systems on soil fertility and carbon storage. Available at http://mro.massey.ac.nz/handle/10179/7180 (2015).
69. Xie, Z. *et al.* Impact of biochar application on nitrogen nutrition of rice, greenhouse-gas emissions and soil organic carbon dynamics in two paddy soils of China. *Plant Soil* **370,** 527–540 (2013).
70. Yamato, M., Okimori, Y., Wibowo, I. F., Anshori, S. & Ogawa, M. Effects of the application of charred bark of Acacia mangium on the yield of maize, cowpea and peanut, and soil chemical properties in South Sumatra, Indonesia. *Soil Sci. Plant Nutr.* **52,** 489–495 (2006).
71. Yeboah, E. *et al.* Improving soil productivity through biochar amendments to soils. *Afr. J. Environ. Sci. Technol.* **3,** 34–41 (2009).
72. Zhang, A. *et al.* Effect of biochar amendment on maize yield and greenhouse gas emissions from a soil organic carbon poor calcareous loamy soil from Central China Plain. *Plant Soil* **351,** 263–275 (2012).
73. Zhang, A. *et al.* Effects of biochar amendment on soil quality, crop yield and greenhouse gas emission in a Chinese rice paddy: a field study of 2 consecutive rice growing cycles. *Field Crops Res.* **127,** 153–160 (2012).
74. Zhang, A. *et al.* Effect of biochar amendment on yield and methane and nitrous oxide emissions from a rice paddy from Tai Lake plain, China. *Agric. Ecosyst. Environ.* **139,** 469–475 (2010).
75. Crane-Droesch, A., Abiven, S., Jeffery, S. & Torn, M. S. Heterogeneous global crop yield response to biochar: a meta-regression analysis. *Environ. Res. Lett.* **8,** 44049 (2013).
76. Lal, R. Enhancing crop yields in the developing countries through restoration of the soil organic carbon pool in agricultural lands. *Land Degrad. Dev.* **17,** 197–209 (2006).
77. Kapkiyai, J. J., Karanja, N. K., Qureshi, J. N., Smithson, P. C. & Woomer, P. L. Soil organic matter and nutrient dynamics in a Kenyan nitisol under long-term fertilizer and organic input management. *Soil Biol. Biochem.* **31,** 1773–1782 (1999).
78. Lal, R. No-tillage effects on soil properties under different crops in western Nigeria. *Soil Sci. Soc. Am. J.* **40,** 762–768 (1976).
79. Diaz-Zorita, M., Buschiazzo, D. E. & Peinemann, N. Soil organic matter and wheat productivity in the semiarid Argentine Pampas. *Agron. J.* **91,** 276–279 (1999).
80. Dıaz-Zorita, M. & Grosso, G. A. Effect of soil texture, organic carbon and water retention on the compactability of soils from the Argentinean pampas. *Soil Tillage Res.* **54,** 121–126 (2000).

81. Petchawee, S. & Chaitep, W. Organic matter management for sustainable agriculture. *Org. Matter Manag. Upl. Syst. Thail. ACIAR Canberra* 21–26 (1995).
82. Shankar, G., Verma, L. & Singh, R. Effect of integrated nutrient management on field and quality of Indian mustard. *Indian J. Agric. Sci.* **72,** 551–552 (2002).
83. Kanchikerimath, M. & Singh, D. Soil organic matter and biological properties after 26 years of maize–wheat–cowpea cropping as affected by manure and fertilization in a Cambisol in semiarid region of India. *Agric. Ecosyst. Environ.* **86,** 155–162 (2001).
84. Samarppuli, I., Ekanayake, A. & Samarppuli, L. Modeling the effects of land degradation on rubber yield. *J. Plant. Crops* **27,** 179–186 (1999).
85. Janzen, H. H., Larney, F. J. & Olson, B. M. Soil quality factors of problem soils in Alberta. in *Proceedings of the Alberta Soil Science Workshop* 17–28 (1992).
86. Olson, B. M. & Janzen, H. H. Influence of topsoil quality parameter on crop yield. in *Proceedings of the Soils and crops workshop, University of Saskatchewan* 137–143 (1992).
87. Clarke, L. *et al.* in *Climate Change 2014: Mitigation of Climate Change. Contribution of Working Group III to the Fifth Assessment Report of the Intergovernmental Panel on Climate Change* (eds Edenhofer, O., R. *et al.*) 413-510 (Cambridge University Press, 2014).
88. FAO. FAOSTAT. Available at http://faostat3.fao.org/ (2016).
89. Bruckner, T. *et al*. in *Climate Change 2014: Mitigation of Climate Change. Contribution of Working Group III to the Fifth Assessment Report of the Intergovernmental Panel on Climate Change* (eds Edenhofer, O., R. *et al.*) 511-597 (Cambridge University Press, 2013).
90. Eisentraut, A., & Brown, A. *Technology Roadmap: Bioenergy for Heat and Power*. (IEA, Paris, 2012).
91. DECC. *Review of the generation costs and deployment potential of renewable electricity technologies in the UK*. (Department of Energy and Climate Change, 2011).
92. Mott MacDonald. *Biomass conversion of a coal plant*. (Committee on Climate Change, London, 2011).
93. Mott MacDonald. *UK Electricity Generation Costs Update*. Available at https://www.gov.uk/government/uploads/system/uploads/attachment_data/file/65716/71-uk-electricity-generation-costs-update-.pdf (2010).
94. IPCC. *Special Report on Renewable Energy Sources and Climate Change Mitigation* (eds Edenhofer, O. et al.) (Cambridge University Press, 2011).
95. Uslu, A., van Stralen, J., Beurskens, L. & Dalla Longa, F. *Utilisation of Sustainable Biomass to Produce Electricity, Heat and Transport Fuels in the EU27-A Model Based Analysis of Biomass Use for 2020 and 2030*. (Energy Research Center of the Netherlands, 2012).
